# Early Development of Hypothalamic Neurons Expressing Proopiomelanocortin Peptides, Neuropeptide Y, and Kisspeptin in Fetal Rhesus Macaques

**DOI:** 10.1523/ENEURO.0087-25.2025

**Published:** 2025-06-26

**Authors:** Oline K. Rønnekleiv, Martha A. Bosch

**Affiliations:** ^1^Department of Chemical Physiology and Biochemistry, Oregon Health and Science University, Portland, Oregon 97239; ^2^Division of Neuroscience, Oregon National Primate Research Center, Oregon Health and Science University, Beaverton, Oregon 97006

## Abstract

We have documented the early embryonic development of hypothalamic neurons expressing β-endorphin, α-melanocyte–stimulating hormone, neuropeptide Y, and kisspeptin in rhesus macaques, an animal model that is very similar to humans. Neurons expressing both β-End and αMSH are the first to develop and are initially located in the lateral basal hypothalamus (LBH) as early as day 32–34 of gestation. By day 45 of gestation, these neurons have migrated into the medial basal hypothalamic (MBH) area as their final destination. NPY neurons within the ARH develop later and first appear at day 44 of fetal life, at which time a cluster of neurons is present within the ARH–MBH area. NPY neurons continue to be expressed within the ARH area at all of the later fetal ages analyzed. Similarly, kisspeptin neurons develop later compared with β-End, although only a few cells are present in the ARH by day 44 of gestation, at which time kisspeptin is also expressed in the developing anterior lobe of the pituitary. By day 70 of gestation, the rostral to caudal distribution and cell size of Kiss1 neurons within the MBH are similar in females and males. In addition, Kiss1 fibers are also expressed in the POA by day 70. By day 130 of gestation, Kiss1 neurons exhibit a wider dorsal and lateral distribution within the MBH, with highly increased fiber distribution. Therefore, the development of these neurons is much earlier than what had been described previously for αMSH and NPY in primates.

## Graphical abstract

**Figure:**
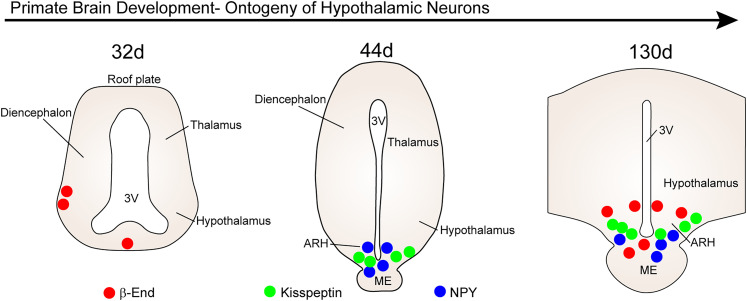
Visual Abstract

## Significance Statement

The arcuate nucleus of the hypothalamus expresses β-End/αMSH, NPY, and kisspeptin neurons, which together are essential for feeding, metabolism, puberty, and fertility. The early development of these neurons in rhesus macaques, which have a long gestation period similar to humans, is not known. Here we show that these neurons start developing within the hypothalamus between day 32 and 44 of gestation and are almost fully developed by midgestation in rhesus macaques. Given that a maternal high-fat diet during pregnancy in primates has been shown to affect melanocortin expression and metabolic health in the mother's offspring, it would be important to know the birthdate and in utero development of these critical neurons in order to prevent postnatal health deficiencies.

## Introduction

Humans have a gestation period of approximately 9 months, whereas nonhuman primates, including rhesus macaques and Japanese macaques, have a gestation period of approximately 166–175 d (Rønnekleiv and Resko, 1990; Grayson et al., 2006). Recently, based on a detailed analysis of the gestation length in rhesus monkeys, Coe and Lubach (2021) concluded that the mean duration from mating to delivery in this primate species is 168.8 d. The early development in primates of hypothalamic neurons involved in feeding and metabolism, such as neuropeptide Y (NPY) and proopiomelanocortin (POMC), is not well known, although the later stage development of some of these neurons has been described (Grayson et al., 2006). Thus, it is known that NPY can be detected in the fetal brain of Japanese macaques starting at embryonic day 100 (E100; Table 1 for abbreviations), at which time NPY mRNA is expressed in the arcuate nucleus of the hypothalamus (ARH), paraventricular nucleus of the hypothalamus (PVH), and dorsomedial nucleus of the hypothalamus (DMH; Grayson et al., 2006). In contrast, the expression of the POMC peptide α-melanocyte–stimulating hormone (α-MSH) in the arcuate nucleus appeared to be minimal at E100 but showed moderate expression at E170 in the Japanese macaque (Grayson et al., 2006).

**Table 1.** Abbreviations

3V	Third ventricle
αMSH	α-Melanocyte stimulating hormone
ACTH	Adrenocorticotropic hormone
AL	Anterior lobe of the pituitary
AR	Androgen receptor
ARH	Arcuate nucleus of the hypothalamus
ARH–MBH	Arcuate nucleus of the hypothalamus–medial basal hypothalamus
ARH/ME	Arcuate nucleus of the hypothalamus/median eminence
AVPV/PeN	Anteroventral periventricular/periventricular nucleus
BH	Basal hypothalamus
BRDU	Bromodeoxyuridine
β-End	β-Endorphin
cARH	Caudal arcuate nucleus of the hypothalamus
Di	Diencephalon
DMH	Dorsomedial nucleus of the hypothalamus
E	Embryonic
E2	17β-Estradiol
ER	Estrogen receptor
F	Female
GnRH	Gonadotropin-releasing hormone
GnRH/LH	Gonadotropin-releasing hormone/luteinizing hormone
GPR54	G-protein-coupled receptor 54 or kisspeptin receptor
IL	Intermediate lobe of the pituitary
IR- β-End	Immunoreactive β-End
IR-Kiss1	Immunoreactive kisspeptin
IR-NPY	Immunoreactive neuropeptide Y
Kiss1	kisspeptin
Kiss1^ARH^	Arcuate kisspeptin neurons
LBH	Lateral basal hypothalamus
M	Male
mARH	Middle arcuate nucleus of the hypothalamus
MBH	Medial basal hypothalamus
ME	Median eminence
NL	Neural lobe of the pituitary
NPY	Neuropeptide Y
PB	Phosphate buffer
PBS	Phosphate-buffered saline
PCR	Polymerase chain reaction
Pit	Pituitary
PND	Postnatal days
POA	Preoptic area
POMC	Proopiomelanocortin
pvPOA	Periventricular preoptic area
PVH	Paraventricular nucleus of the hypothalamus
rARH	Rostral arcuate nucleus of the hypothalamus
RIA	Radioimmunoassay
VMH	Ventromedial hypothalamus

The location and development of POMC peptides including β-endorphin (β-End) and αMSH have not been documented in rhesus macaques or humans. However, recent studies on human embryonic brain samples have described the transcriptional profile of thousands of cells during early development in some detail (Fang et al., 2010; Zeng et al., 2023). The organization of the human hypothalamus and the later expression of several neuropeptides including NPY have also been described in the human infundibular (arcuate) nucleus beginning at gestational week 21 (Koutcherov et al., 2003).

In comparison, much more is known about the development of hypothalamic neurons important for reproduction and metabolism in different rodent species. Thus, it has been shown that POMC mRNA detected by in situ hybridization and adrenocorticotropic hormone (ACTH)-immunoreactivity appear simultaneously at embryonic day 10.5 (of a 21 d gestation period) in the mouse ventral diencephalon (Elkabes et al., 1989). More recently, it was discovered that POMC-expressing progenitors in mice differentiate not only to POMC-expressing neurons but also to NPY and arcuate kisspeptin (Kiss1^ARH^) neurons that no longer express POMC (Padilla et al., 2010; Sanz et al., 2015). This would be an indication that POMC-expressing neurons are among the first to develop in the mouse ventral diencephalon, and some of these early POMC neurons are then converted to NPY and Kiss1^ARH^ neurons.

In mice, NPY transcripts are first detected in the presumptive ARH at E14.5 (Padilla et al., 2010), whereas the expression of mRNA encoding for Kiss1 and the Kiss1 receptor (GPR 54) can be observed at E13 in the ARH by using in situ hybridization (Knoll et al., 2013).

Based on bromodeoxyuridine (BRDU) labeling in developing rats, the period of neurogenesis of Kiss1^ARH^ neurons in this species begins between E12.5 and E13.5, reaches a peak at E15.5, but is not completed by E17. In support of these findings, the investigators found that a few immunoreactive Kiss1 cells were first detected in the rat ARH starting at E14.5 in both females and males, which continued to increase until E18.5, at which time the number of Kiss1^ARH^ neurons decreased and was substantially reduced at the time of birth at 21 d (Desroziers et al., 2012). Therefore, it was concluded that Kiss1^ARH^ neurons are born locally during an extended embryonic period and do not migrate from other brain regions (Desroziers et al., 2012). In addition to mRNA, a few kisspeptin-immunoreactive (IR-Kiss1) cells were detected just dorsal to the optic chiasm in the POA at E18.5 in rats (Desroziers et al., 2012). However, according to others, kisspeptin neurons in the POA develop later and are not detected in the POA before postnatal days (PND) 10–11 in rats (Semaan et al., 2010; Cao and Patisaul, 2011). Essentially nothing is known about the origin of these rostral kisspeptin neurons localized in the anteroventral periventricular/periventricular nucleus (AVPV/PeN), but these neurons are not derived (differentiated) from POMC-expressing precursors (Sanz et al., 2015).

Given that these hypothalamic neuropeptides are important for fertility and metabolism and very little is known about their development in primates, studies were initiated to explore the early time course and location of the POMC peptides, β-End, and αMSH, as well as NPY and Kiss1 neurons at different embryonic times in fetal rhesus macaques, since their development is similar to that in human (Stouffer and Woodruff, 2017).

## Materials and Methods

### Animals

All animal procedures were approved by our Institutional Animal Care and Use Committee and according to US National Institutes of Health Guidelines on the ethical use of animals. Adult female rhesus monkeys (*Macaca mulatta*) were paired with fertile males for 3 d beginning on day 9–18 of their menstrual cycle, based on an analysis of their previous menstrual cycle lengths. Pregnancy was determined by radioimmunoassay (RIA) analysis of estrogen (>100 pg/ml) and progesterone (>2.5 ng/ml) in blood samples obtained at days 13–17 after pairing (Hess et al., 1981). The second day of pairing was chosen as the day of conception; gestation times were calculated from that point. At various times during gestation, beginning at day 32, the fetuses were delivered by cesarean section, and the brains were prepared for analysis of various hypothalamic neurohormones using immunohistochemistry for kisspeptin (Kiss1), NPY, and the POMC peptides β-End and αMSH. In addition, the fetal liver was often harvested and snap frozen without fixation to be used for sex determination in the younger animals.

In humans, prenatal life is divided into two phases: embryonic and fetal. The embryonic period occupies the first 8 postovulatory weeks. The fetal period extends from 8 weeks to birth (O'Rahilly and Muller, 1992). Since a similar definition has not been described in nonhuman primates, we have used the term fetal for all animals used in this study (Table 2).

**Table 2.** Physiological and anatomical data from fetuses used to determine the ontogeny of hypothalamic neurons

Animal #	Sex	Fetal age (days)	BW (g)	C–R length (cm)	Head circ (cm)
412	F	32	0.127	0.90	
408	M	34		1.55	
411	M	35	0.300	1.40	
415	M	37	0.581	1.69	
286	F	40	0.510	1.60	2.30
414		40	0.638	1.95	
245	F	41			
246	F	41	0.800		
327	M	44	1.429	2.29	3.30
256	M	45	1.000	2.15	2.90
413	M	47	4.370	2.95	3.65
249	F	59	7.300	4.50	5.50
248	M	59	10.920	5.19	6.20
257	M	60	10.360	5.22	5.50
243	F	61	8.800	4.70	5.85
223	M	63	10.300	5.10	6.40
330	F	69	22.850	7.64	7.40
351	F	70	27.510	7.96	8.20
354	F	70	21.700	7.19	7.80
321	M	70	22.410	7.29	7.50
381	M	70	35.624	8.29	8.50
382	M	70	31.950	8.05	8.15
386	F	129	297.300	17.10	18.30
365	F	130	289.400	16.82	17.10
370	F	130	269.320	16.34	18.20
378	F	130	287.000	16.80	17.50
385	F	130	289.700	17.00	17.90
383	M	129	319.500	16.75	17.50
375	M	130	355.900	17.40	18.50
390	M	130	359.190	17.24	18.60

Abbreviations: F, female; M, male; BW, body weight; C–R length, crown to rump length; Head circ, head circumference. Estimation statistics of the difference in body weight between males and females at days 129–130 is shown in Extended Data Table 2-1.

10.1523/ENEURO.0087-25.2025.t2-1 Table 2-1 Estimation statistics comparing body weight between females and males at 130 d gestation. Download Table 2-1, DOC file.

Physiological and anatomical data from fetuses used in this study are presented in Table 2. This included body weight, crown–rump length, and head circumference for most of the animals. We had one each from days 32, 34, 35, and 37; two day 40; two day 41; one day 44; two day 45; one day 47; two day 59; one day 60; one day 61; one day 63; one day 69; five day 70 (three males and two females); two day 129 (one female and one male); and six day 130 fetuses (four females and two males). These were mostly control animals from previously funded studies. Brain tissues and sections were stored frozen until used for this developmental study.

### Fetal sex determination

DNA was extracted from frozen liver tissue or from fixed tissue sections mounted on glass slides to determine the sex of the younger fetuses. Extraction reagents from the Extract-N-Amp kit (Sigma-Aldrich) were used to extract DNA from the liver tissues with a slight modification to the manufacturer’s protocol. Briefly, 100 μl of extraction solution and 25 μl of tissue preparation solution were added to a tube containing 30–50 mg of liver tissue, and a pestle was used to gently disrupt the tissue. The sample was incubated in a 37°C water bath for 20 min and then incubated in a 95°C heat block for 4 min. Immediately following the incubations, 100 μl of neutralization buffer was added to the sample. The samples were then stored at 4°C. DNA extraction from fixed tissue on slides was performed on six to nine 20 μm sections according to an established protocol (Campos and Gilbert, 2012). Briefly, tissue sections were removed from the slides with a single-edge blade, placed in 0.5 ml of digestion buffer (0.1 M NaOH, 1% SDS, pH 12), and heated in a heat block at 100°C for 40 min. A phenol-chloroform extraction followed by an isopropanol and sodium acetate precipitation produced a DNA pellet that was reconstituted in distilled water and quantified on the NanoDrop. Primers were designed to target the X and Y chromosomes and are as follows: X-chromosome primers, accession number DQ037728, forward primer 5′ TGCTACCTCAGGTGGACAACAAGG 3′, reverse primer 5′ CTCGACACTGGCAGTGCTGTTAGG 3′, 127 bp product (Villesen and Fredsted, 2006); Y-chromosome primers; accession number XR_003727107, forward primer 5′ GTCACCTCAGGTGGACAACA 3′, reverse primer 5′ TGCTTGTTTCAGGCACCAAG 3′, 85 bp product. PCR was performed on 3–5 μl of extracted DNA in a 30 μl reaction containing 6 μl 5× GoTaq buffer (Promega), 1.5 mM MgCl_2_ (X primers), 2.0 mM MgCl_2_ (Y primers), 0.2 mM dNTPs, 0.3 μM forward and reverse primers, 2 units (U) of GoTaq polymerase (Promega), and 0.22 μg TaqStart antibody (Takara). PCR protocol: 94°C for 3 min followed by 35 cycles of 94°C for 30 s, 57°C (X primers) and 55°C (Y primers) for 30 s, and 72°C for 30 s followed by a 72°C extension for 5 min. PCR products were visualized with ethidium bromide on a 2% agarose gel. Samples from older fetuses where the sex could be determined visually were included as positive controls.

### Tissue preparation

The whole fetus or just the head was removed from the E32, E34, E35, and E37 animals and placed in paraformaldehyde fixative solution (4% in 0.03M Sorensen's phosphate buffer, pH 7.4) for 4–5 h and then soaked overnight in 30% sucrose buffer solution, frozen at −55°C. At these ages, the developing skull was pierced to allow fixation penetration to the brain. The whole fetus or head was cut sagittally or coronally into 20–25 μm sections on a cryostat, and the sections were thaw-mounted on Superfrost Plus glass slides (Thermo Fisher Scientific). In E40–E47 animals, the brain was removed from the developing skull and fixed in paraformaldehyde solution as described above for ∼5 h. In E59 and older fetuses, the preoptic and basal hypothalamic areas were dissected and fixed by immersion in paraformaldehyde solution for 5–7 h. Thereafter, all tissues were soaked overnight in 30% sucrose buffer solution, frozen in OCT, and stored at −80°C until sectioned on a cryostat.

### Immunohistochemistry

The sections mounted on glass slides were rinsed in phosphate buffer (PB; 0.1 M, pH 7.4) for at least 30 min. Next, sections were incubated with 5% normal serum (corresponding to the host for the secondary antiserum) in 0.3% Triton X-100 in PBS for 30 min, rinsed in PB, and then incubated for ∼45 h at 4°C with rabbit polyclonal antisera against the following peptides: β-endorphin (β-End; 1:5,000; Dave et al., 1985), α-melanocyte stimulating hormone (α-MSH; 1:5,000; Dave et al., 1985), neuropeptide Y (NPY; from 1:2,500 to 1:5,000; Sabatino et al., 1987), and kisspeptin (KISS1 564; 1:2,500; Franceschini et al., 2006). In addition, select brain sections were also reacted with an antibody against gonadotropin-releasing hormone (GnRH; EL14; Ellinwood et al., 1985), a very selective conformational antibody against GnRH to help identify hypothalamic nuclei in early embryonic animals. This was helpful since the distribution of GnRH has been described in detail in the embryonic monkey brain (Rønnekleiv and Resko, 1990; Quanbeck et al., 1997). The specificity of these antisera in fetal monkey brains was documented as follows: The diluted primary antisera (1.5 ml) were incubated overnight with 10 μg of the corresponding peptide (see below) before being added to brain sections, a procedure that eliminated all specific staining. After a 30 min wash in PB, sections were first incubated for 2 h at room temperature with biotinylated donkey anti-rabbit gamma globulin (IgG; 1:500; Jackson ImmunoResearch catalog #711-065-152, RRID:AB_2340593) and next with streptavidin Alexa 488 (1:2,500; Jackson ImmunoResearch catalog #016540084, RRID:AB_2337249) or streptavidin Alexa 594 (1:2,500; Jackson ImmunoResearch catalog #016580084, RRID:AB_2337250) for 3 h. Both the primary and secondary antibodies were diluted in Tris-phosphate buffer: Tris base [Tris(hydroxymethyl)aminomethane, 41 mM; Na_2_HPO_4_, 8.5 mM; KH_2_PO_4_, 3.5 mM; NaCl,120 mM; adjusted to pH 7.6] containing 0.7% seaweed gelatin (Sigma-Aldrich), 0.5% Triton X-100, and 3% bovine serum albumin (BSA; Sigma-Aldrich). Following a final wash overnight, slides were coverslipped with gelvatol containing the antifading agent, 1,4-diazabicyclo(2,2)octane (DABCO; Cold Spring Harbor Protocols, 2006).

### Peptides used for immunoabsorption

Beta-endorphin peptide sequence (human; Abbiotec): YGGFMTSEKSQTPLVTLFKNAIIKNAYKKGE.

NPY peptide sequence (human, rat; Tocris Bioscience): YPSKPDNPGEDAPAEDMARYYSALRHYINLITRQRY.

Kisspeptin peptide sequence (Phoenix Pharmaceuticals): YNWNSFGLRF

### Imaging

Photomicrographs of antiserum labeling were acquired using a Nikon E800 fluorescent microscope (Eclipse E800; Nikon Instruments) equipped with a fiber illuminator (Intensilight C-HGFI; Nikon Instruments) and a high-definition digital microscope camera head (DS_Fi1; Nikon Instruments) interfaced with a PC-based camera controller (DS-U3; Nikon Instruments). Following photography, only brightness and contrast were used to modify images.

### Data analysis

Low-power images were used to determine the distribution of the neuropeptides, and high-power images were used to better illustrate the neuronal cell body size and shape at the different developmental stages. Cell size determination: Photographic images with scale bars, stained for kisspeptin in the ARH from day 70 and day 130 females and males, were analyzed for cell size. Viewing images at 40× magnification in Adobe Illustrator, individual cells were measured based on the scale bar generated by the Nikon camera system. Lines were drawn spanning the length and width of each cell using the Illustrator line tool, and the length of the lines in micrometers was determined based on the scale bar.

Body weight analysis: Differences in body weight were measured in day 70 and day 130 fetuses, and the results were analyzed using the unpaired *t* test as well as estimation statistics (Bernard, 2019; Ho et al., 2019).

## Results

In this study, we were interested in exploring the early development of hypothalamic neurons in rhesus macaques including those expressing the POMC peptides β-End and α-MSH, as well as neuropeptide Y (NPY) and kisspeptin (Kiss1) which are involved in metabolism and fertility. Previous studies have documented that gonadotropin-releasing hormone (GnRH) neurons are developed in the nose and migrate to the olfactory area of the brain starting at days 32–36 of gestation (Rønnekleiv and Resko, 1990; Quanbeck et al., 1997). These neurons, identified using different GnRH antibodies including the EL14 and LR-1 antibodies, continue to migrate further caudal toward the BH area where they are first expressed at days 47–51 of gestation (Rønnekleiv and Resko, 1990; Quanbeck et al., 1997). Therefore, we anticipated that β-End, αMSH, NPY, and Kiss1 neurons might be first expressed in the hypothalamus at about the same time in development.

Physiological and anatomical data from rhesus macaque fetuses used in this study are presented in Table 2. Our youngest fetus was 32 d and the oldest 130 d. Body weight increased gradually with age, and there were no apparent differences between males and females. However, by day 129–130 of gestation, males (344.9 ± 12.72; *n* = 3) were significantly heavier compared with females (286.5 ± 4.64; *n* = 5; unpaired *t* test, *t*_6_ = 5.226, *p* = 0.002; Table 2; Extended Data Table 2-1).

As stated in Materials and Methods, we needed to measure the X and Y chromosomes in order to determine the sex of the younger fetuses. For most of the fetuses, we had frozen liver for this purpose. However, in some instances, DNA from other tissues, including brain sections mounted on slides, was also used to obtain DNA for sex determination.

**Figure 1.**
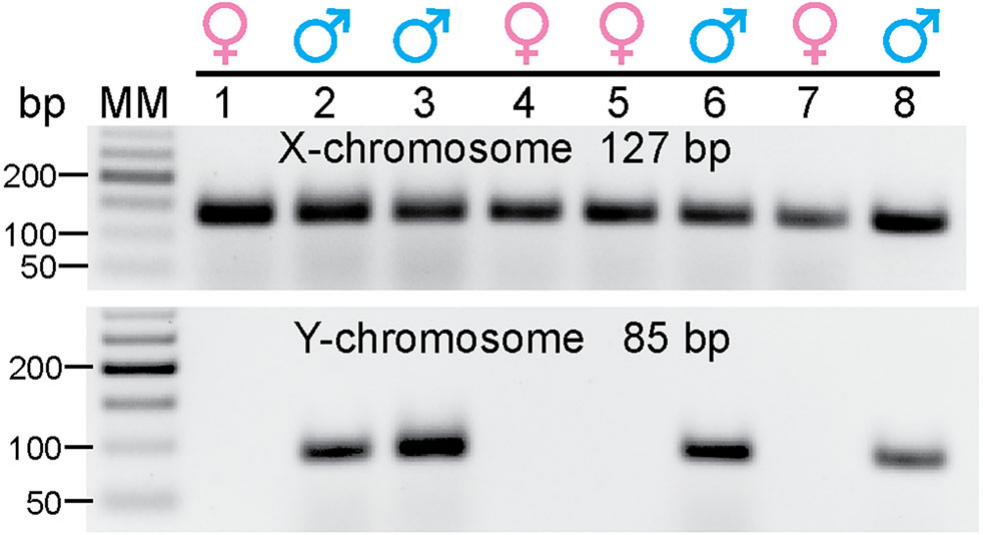
Sex determination of 40–45 d fetuses. DNA was extracted from liver or brain tissue sections as described in Materials and Methods. PCR was performed using primers for the X and Y chromosomes (see Materials and Methods). A representative gel illustrates six young fetuses (40–45 d; Lanes 1–6) and two older control fetuses (70 d; Lanes 7–8). Bp, base pairs. MM, molecular markers, also called DNA ladder.

DNA was extracted from fetal liver or other tissues and used to determine the sex of the younger fetuses. Samples from older fetuses where the sex could be determined visually were included as positive controls. As expected, all samples produced a band for the X chromosome and only the male samples produced a band for the Y chromosome (Fig. 1).

### Development of proopiomelanocortin (POMC) peptides

**Figure 2.**
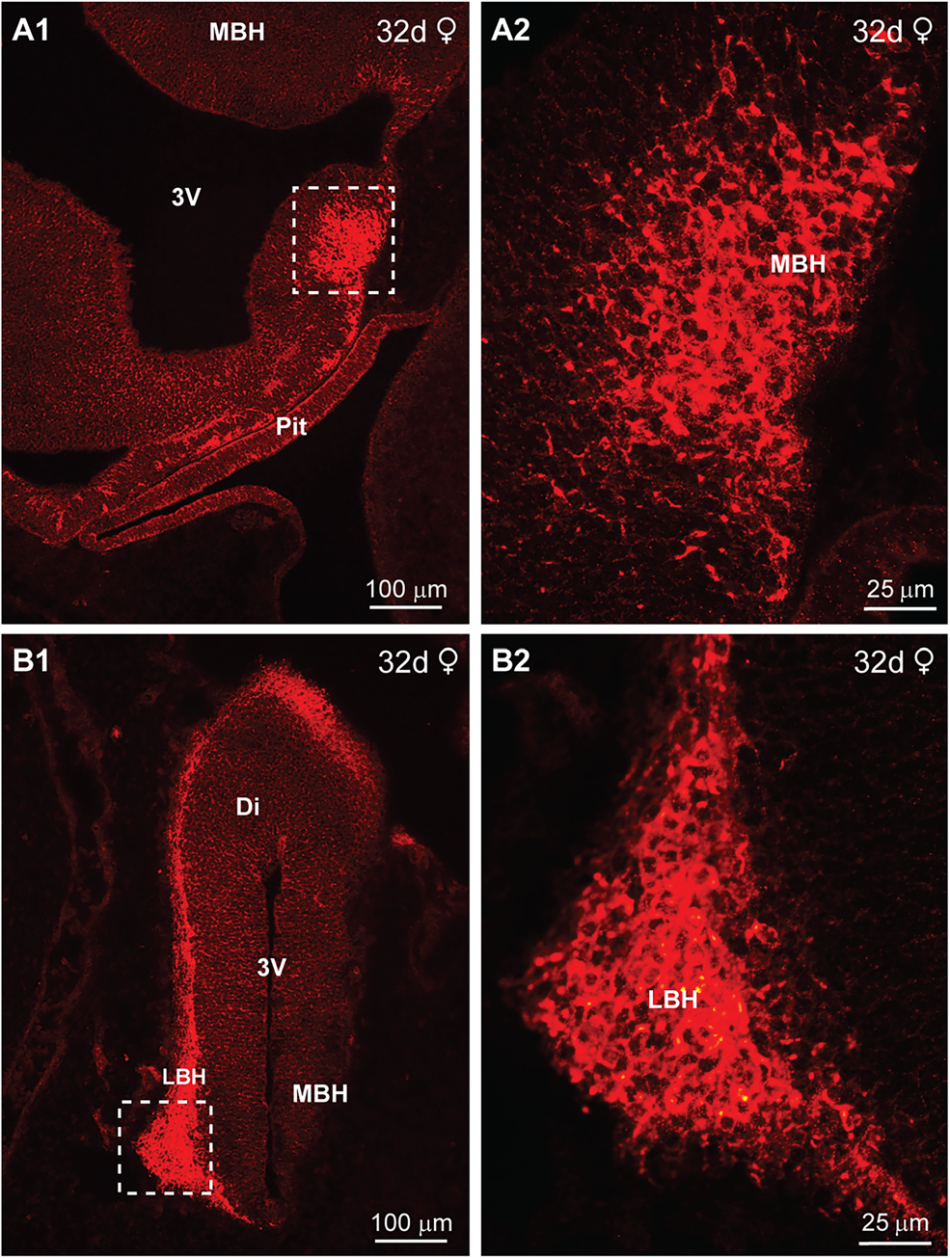
Immunoreactive-βEnd expression in the fetal macaque brain at day 32 of gestation*.* Fluorescent images of coronal sections through the developing diencephalon (Di) and pituitary (Pit or Ratke's pouch) of a 32 d female fetus illustrating immunoreactive βEnd (IR-βEnd) within the medial basal hypothalamus (MBH) and lateral basal hypothalamus (LBH) and along the surface of the diencephalon (Di; ***A1*–*B1***). The stippled areas in ***A1*** and ***B1*** are shown at higher magnification to illustrate β-End cells and fibers primarily within the developing hypothalamus (***A2***, ***B2***). Note that β-End is not expressed in the developing pituitary at this early stage of gestation (***A1***). Different scale bars are shown for all images.

**Figure 3.**
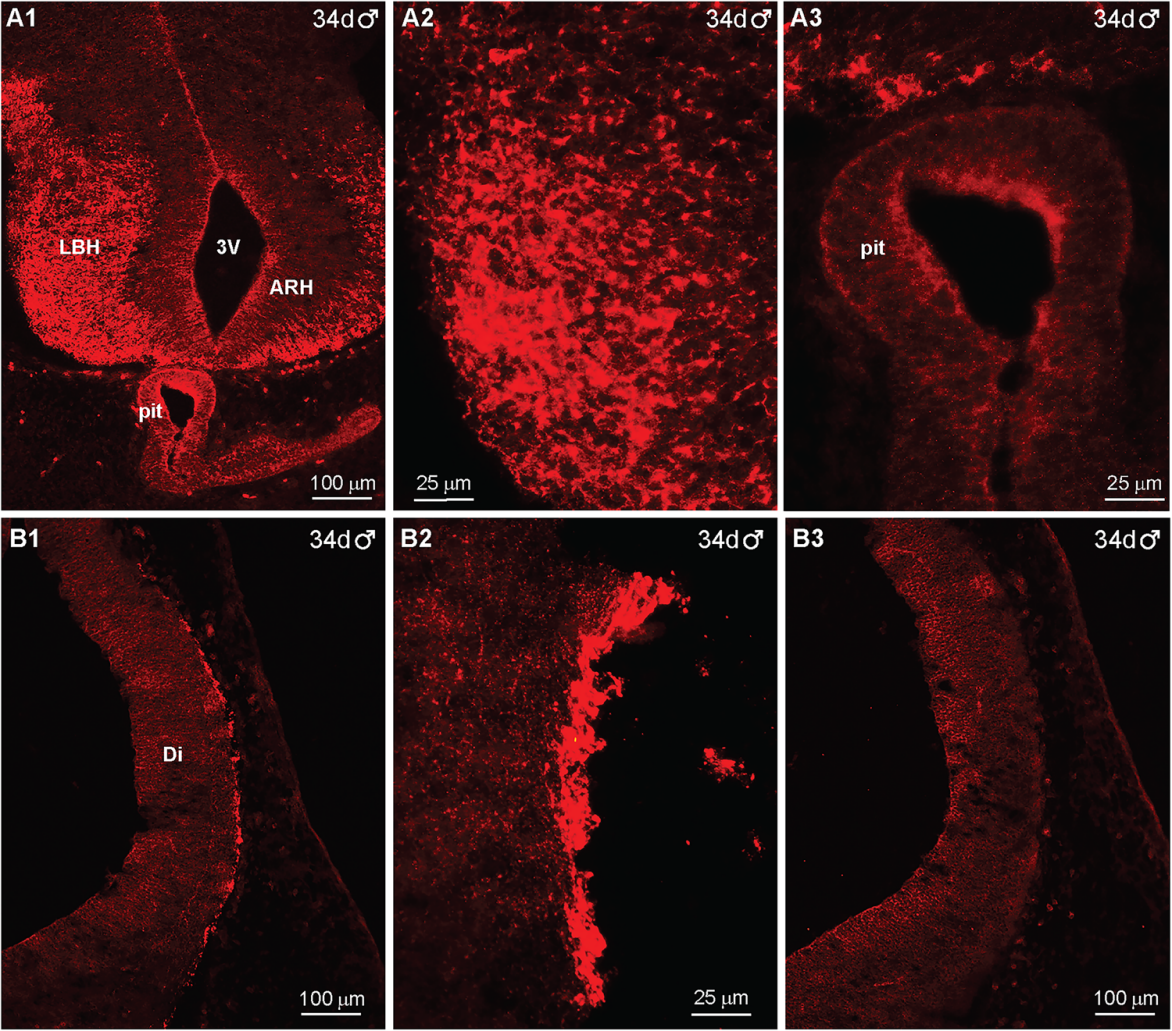
Immunoreactive-βEnd expression in the fetal macaque brain at day 34 of gestation*.* Fluorescent images of coronal sections through the hypothalamic area and pituitary (***A1*–*A3***) and dorsal diencephalon (Di; ***B1*–*B3***) of a 34 d male fetus illustrating immunoreactive-βEnd (IR-βEnd) in the lateral basal hypothalamus (LBH) and the developing pituitary (Pit). The image in ***A2*** shows a high-power image of IR-βEnd in ***A1***. The high-power view of the pituitary in ***A3*** demonstrates a lack of IR-βEnd in the pituitary at this time in gestation. The low-power view in ***B1*** illustrates IR-βEnd along the dorsal diencephalon (Di), with a high-power image in ***B2***, which shows IR-βEnd cells and fibers along the periphery. The section adjacent to ***B1***, shown in ***B3***, was reacted with the βEnd antiserum preabsorbed with the corresponding POMC peptide, which eliminated the IR-βEnd. In addition, a high-power image of βEnd and αMSH is also shown in Extended Data Figure 3-1. Different scale bars are shown for all images (***A1*–*B3***).

The POMC peptide β-endorphin (β-End) was first detected in the fetal monkey hypothalamus at day 32 of gestation in a female. At this stage, only a small area of immunoreactive β-End (IR-βEnd) cells and fibers was expressed in the more ventral basal hypothalamus (BH) with most of the area filled with a large ventricular cavity (Figs. 2*A1*,*A2*, 22). Also, the pituitary did not express IR-βEnd at this point in development (Fig. 2*A1*). Further caudal in the BH, a cluster of IR-βEnd cells was located ventrolaterally in the hypothalamus (Fig. *2B1*,*B2*). The developing neurons were “clumped” together, had irregular shapes at higher magnification and therefore looked immature (Fig. 2*A2*–*B2*). IR-βEnd cells and fibers could also be traced along the lateral surface of the diencephalon (Fig. 2*B1*). However, only 2 d later, by day 34 of gestation in a male, the hypothalamus was more developed, and the third ventricle was substantially reduced (Figs. 3*A1*, 22). At this stage, IR-βEnd (cells and fibers) were expressed primarily in the ventrolateral BH but were not expressed in the pituitary (Fig. 3*A1*–*A3*). In addition, IR-βEnd cells and fibers were also present along the outer surface of the fetal diencephalon (Fig. 3*B1*,*B2*). The immunoreactive staining was specific for β-End given that it was eliminated when adjacent sections were reacted with antiserum preabsorbed with the β-End sequence of the POMC peptide (Fig. 3*B3*); see Materials and Methods for peptide sequence. It is, however, unclear from where the cells located around the periphery originated. In addition to βEnd (Extended Data Fig. 3-1*A*), the POMC peptide αMSH was also observed in the lateral hypothalamus at day 35 of gestation (Extended Data Fig. 3-1*B*), which is not surprising since these peptides are derived from the same POMC precursor peptide (O'Donohue and Dorsa, 1982; Kiss et al., 1985).

10.1523/ENEURO.0087-25.2025.f3-1 Figure 3-1 *High power image of β-END and αMSH in the lateral BH at day 35 of gestation. * Fluorescent images of coronal sections through the lateral BH (LBH) area (**A, B**) of a 35 day male fetus illustrating that the immunoreactive proopiomelanocortin (POMC) peptides βEnd (**A**) and αMSH (**B**) are both present at this early stage in gestation. Download Figure 3-1, TIF file.

**Figure 4.**
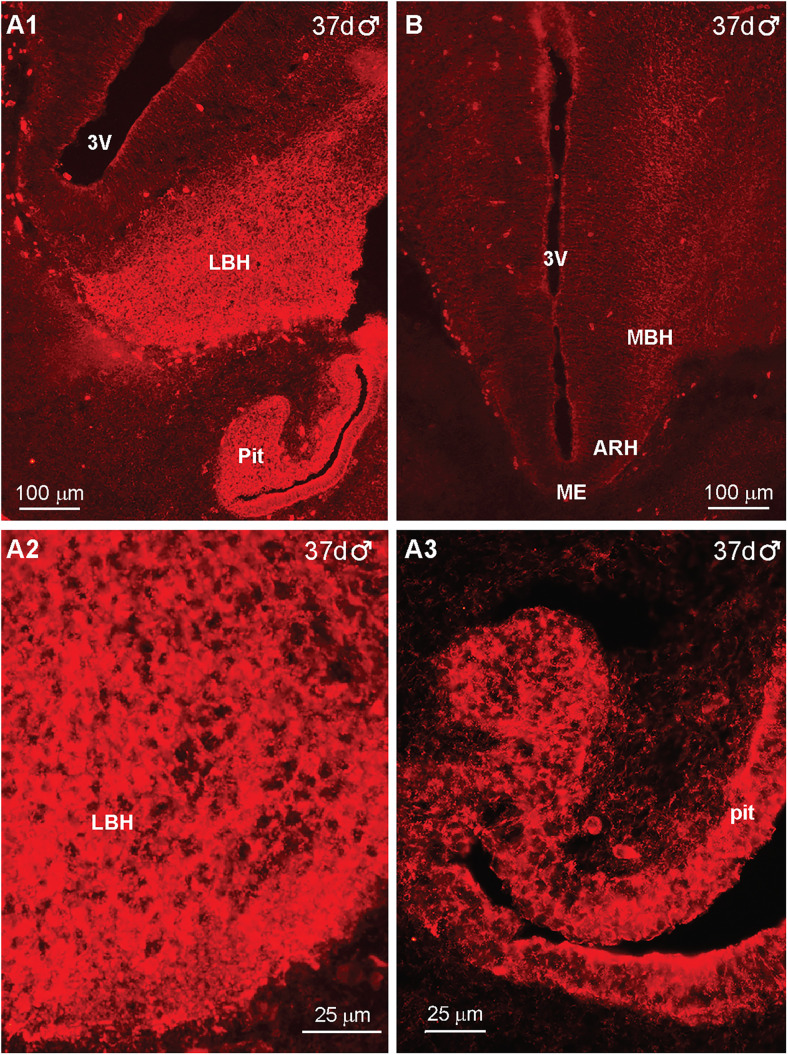
Immunoreactive-βEnd expression in the fetal brain and pituitary at day 37 of gestation*.* Low-power image of IR-βEnd in the hypothalamus and pituitary gland of a day 37 male embryo (Fig. 4*A1*). The corresponding high-power images are illustrated in ***A2*** and ***A3***, respectively. A hypothalamic section reacted with peptide-preabsorbed antiserum, revealing a lack of IR-βEnd (Fig. 4*B*). A similar distribution of IR-βEnd in the lateral BH is also present at day 40 of gestation as shown in Extended Data Figure 4-1. Abbreviations: MBH, medial basal hypothalamus; LBH, lateral basal hypothalamus; Pit, pituitary; 3V, third ventricle. Different scale bars are shown for all images.

In days 37–40 fetuses, IR-βEnd was detected in the ventrolateral BH and also in the pituitary (Rathke's pouch), and this staining was eliminated when reacted with antiserum preabsorbed with the β-End peptide sequence (Fig. 4*A1*,*B*; Extended Data Fig. 4-1). The high-power image (Fig. 4*A2*) illustrates that IR-βEnd cells continue to be highly expressed in the lateral BH (LBH) at this stage in gestation. At this fetal age, IR-βEnd is also present in the developing pituitary (Fig. 4*A3*).

10.1523/ENEURO.0087-25.2025.f4-1 Figure 4-1 ***Immunoreactive-βEnd expression in the fetal brain at day 40 of gestation.*** The fluorescent image illustrates the distribution of IR-βEnd in the lateral basal hypothalamus (LBH) at day 40 of gestation. Abbreviations: ARH, arcuate nucleus of the hypothalamus; ME median eminence; 3V, third ventricle. Scale bar  =  100µm. Download Figure 4-1, TIF file.

Therefore, the two different POMC peptides, β-End and αMSH, seem to be coexpressed in the hypothalamus quite early in gestation in rhesus macaques. In contrast, NPY and kisspeptin were not detected in the BH or pituitary at this early stage in gestation (see below).

**Figure 5.**
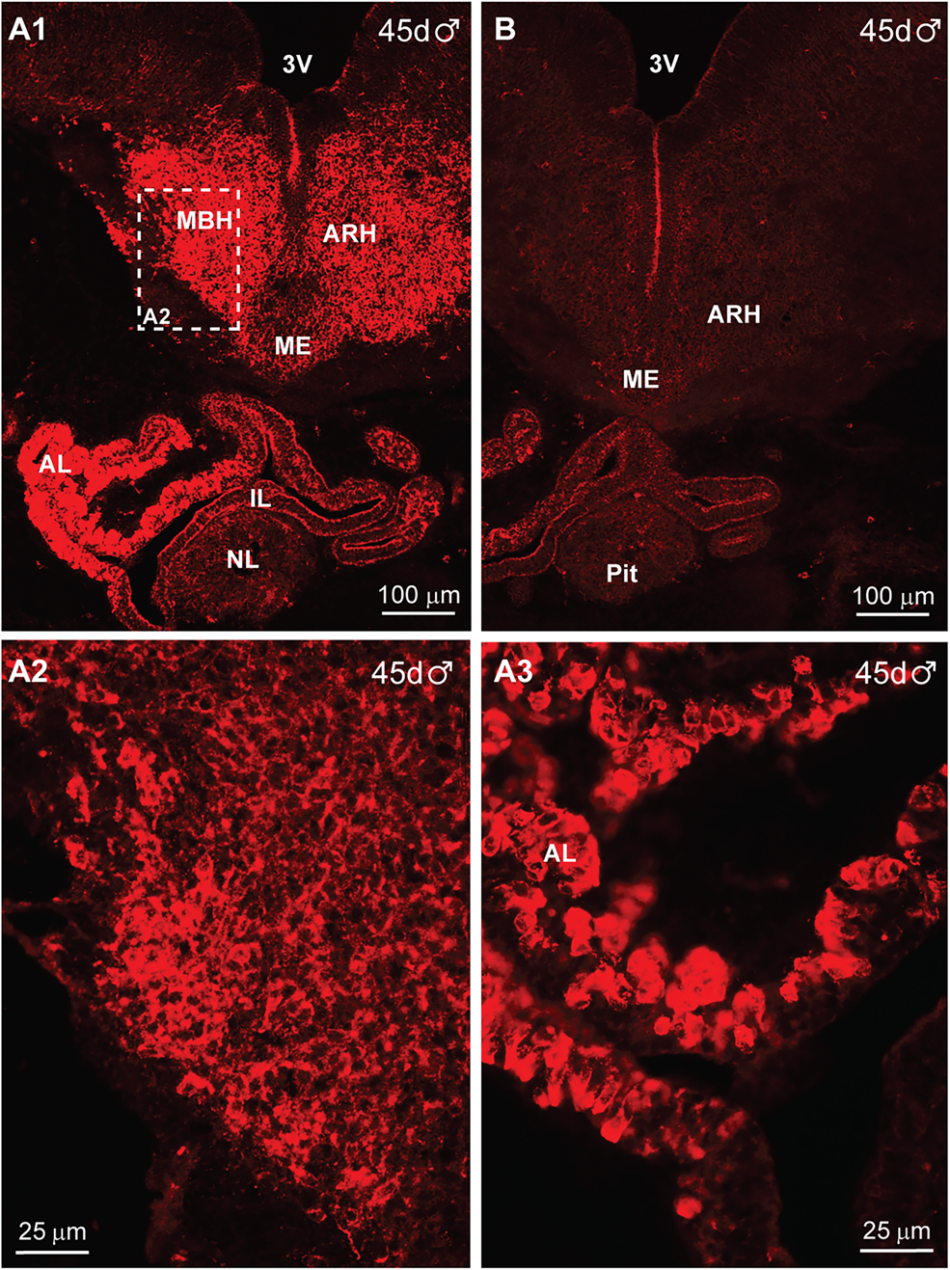
Immunoreactive-βEnd expression in the fetal hypothalamus and pituitary at day 45 of gestation. Low-power fluorescent images of coronal sections through the hypothalamus and pituitary of a day 45 male fetus that were reacted with βEnd antiserum (***A1***) or βEnd antiserum preabsorbed with the corresponding peptide to illustrate the specificity of the data (***B***). The stippled area in ***A1*** is shown at higher magnification in (***A2***). The high-power images in ***A3*** show the βEnd-positive cells in the anterior lobe (AL) of the pituitary gland. A similar hypothalamic and pituitary distribution of IR-αMSH is shown in Extended Data Figure 5-1. In addition, the IR-βEnd or βEnd absorbed with peptide on the surface of the diencephalon is also illustrated in Extended Data Figure 5-1. Abbreviations: MBH, medial basal hypothalamus; ARH, arcuate nucleus of the hypothalamus; ME, median eminence, 3V, third ventricle; IL, intermediate lobe of the pituitary; NL, neural lobe of the pituitary. Different scale bars are shown for all images.

By days 44–45 of gestation, the IR-βEnd staining of cells and fibers was the most extensive throughout the medial basal hypothalamus (MBH) including the ARH (infundibular) nucleus, and the median eminence (ME; Fig. 5*A1*). Of interest is that the pituitary was much more developed at day 45 as compared with day 37 of gestation, in that one could now discern the different lobes of the pituitary, i.e., the anterior, intermediate, and neural lobes (Fig. 5*A1*). However, β-End was only expressed in the anterior lobe at this stage in gestation. Importantly, all staining was eliminated when the adjacent brain–pituitary section was reacted with antiserum preabsorbed with the corresponding β-End POMC peptide (see Materials and Methods; Fig. 5*B*). The high-power images of Figure 5*A1*, as shown in Figure 5*A2* and *A3*, illustrate IR-βEnd neurons and fibers in the ventromedial hypothalamus, as well as β-End cells in the anterior lobe (AL) of the pituitary gland. In addition, IR-αMSH cells were also expressed in the ventromedial BH and the pituitary (Extended Data Fig. 5-1*A*). Also, even at this stage in development, β-End cells and fibers were present along the periphery of the diencephalon, and the β-End staining was specific since it was eliminated when the β-End antiserum was preabsorbed with the corresponding POMC peptide (Extended Data Fig. 5-1*B1*–*B3*).

10.1523/ENEURO.0087-25.2025.f5-1 Figure 5-1 *IR-αMSH and IR-βEnd expression in the fetal brain and pituitary at day 44-45 of gestation.* Fluorescent images of coronal sections through the hypothalamus and pituitary (**A**), and diencephalon (Di) (**B1- B3**) of days 44-45 male fetuses that were reacted with αMSH (**A**) or βEnd antisera (**B1-B2**), respectively, or βEnd antiserum preabsorbed with the corresponding peptide to illustrate the specificity of the data (**B3)**. The different scale bars are shown for all images. Download Figure 5-1, TIF file.

**Figure 6.**
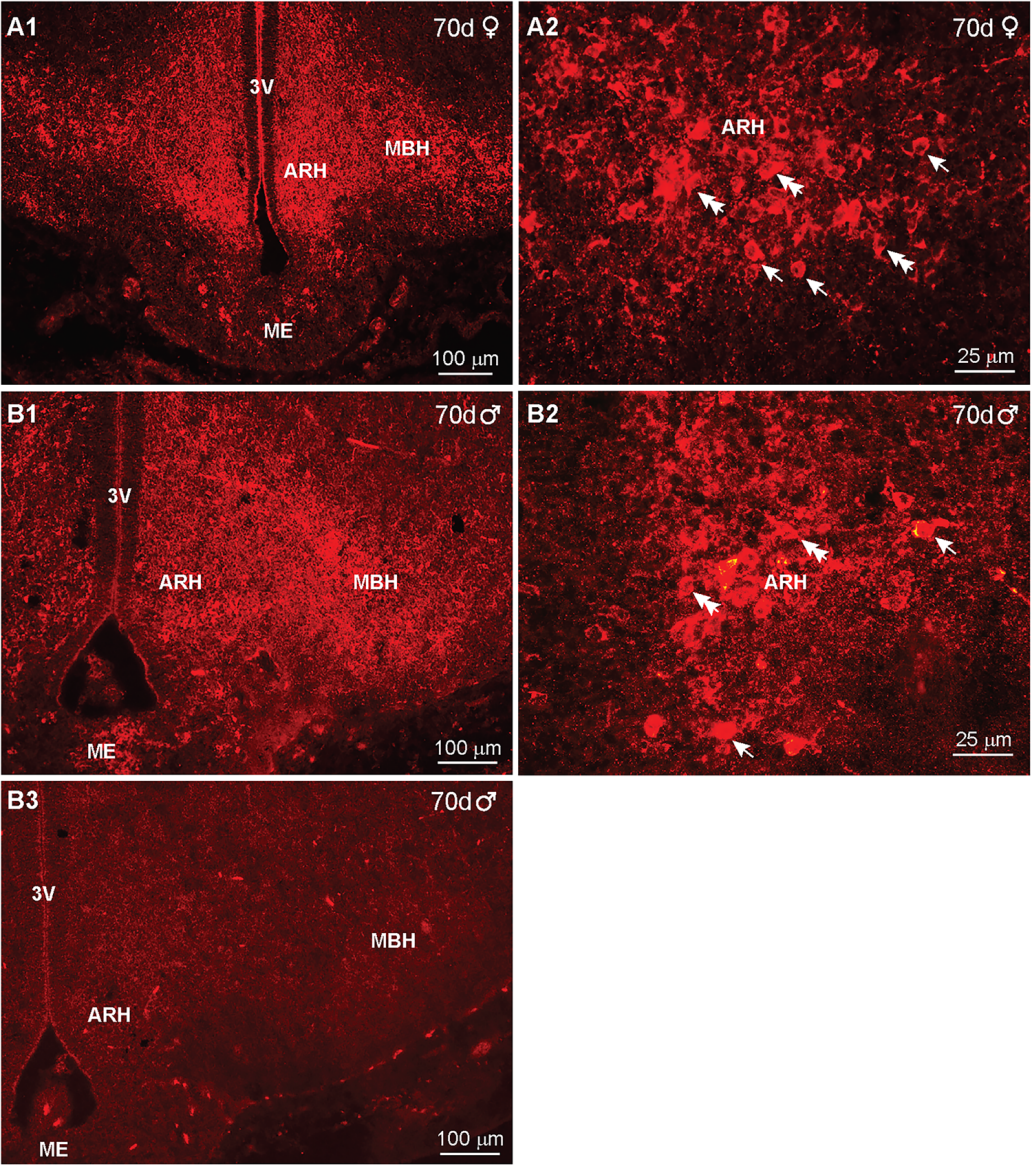
IR-βEnd expression in the fetal hypothalamus at day 70 of gestation. Fluorescent images of coronal sections through the basal hypothalamus of a day 70 female (***A1***, ***A2***) and a day 70 male rhesus macaque fetus (***B1***, ***B2***) illustrating IR-βEnd within the ARH and MBH area. High-power images of ***A1*** and ***B1*** are shown in ***A2*** and ***B2***, respectively. In addition, an adjacent section to ***B1*** was reacted with peptide-preabsorbed β-End antiserum, which eliminated IR-βEnd staining (***B3***). A similar basal hypothalamic distribution of αMSH is shown in Extended Data Figure 6-1. Abbreviations: ARH, arcuate nucleus hypothalamus; MBH, medial basal hypothalamus; ME, median eminence. The different scale bars (100 or 25 μm) indicate the degree of magnification. Arrows point to round cells and double arrows to fusiform cells.

By day 70 of gestation, the MBH area was increased, and the density of IR-βEnd cells and fibers was reduced in some areas and increased in others in both females and males. IR-βEnd cells were located both in the ARH and more lateral in the MBH areas in both females and males (Fig. 6*A1*,*B1*, respectively). High-power image of the arcuate β-End cells shows primarily simple round to fusiform cells in both females and males (Fig. 6*A2*,*B2*). As noted above, all immunoreactive staining (of cells and fibers) was eliminated when BH sections were reacted with peptide-preabsorbed antiserum as shown here in the 70 d male fetuses (Fig. 6*B3*). In contrast to days 44–45 of gestation, fibers and cells along the periphery of the diencephalon appeared not to be present in day 70 and older fetuses. Similar to β-End the POMC peptide αMSH exhibited an almost identical immunoreactive distribution within the ARH/MBH area (Extended Data Fig. 6-1*A1*). Also, the high-power image illustrates primarily round to fusiform cells marked by arrows or double arrows, respectively (Extended Data Fig. 6-1*A2*).

10.1523/ENEURO.0087-25.2025.f6-1 Figure 6-1 *IR-αMSH expression in the fetal hypothalamus at day 70 of gestation.* Fluorescent images of coronal sections through the BH of a day 70 female illustrating IR-αMSH within the hypothalamic area (**A1**). A high-power image is shown in (**A2**). The different scale-bars (100µm or 25µm) indicate the degree of magnification. Arrows point to round cells and double arrows to more fusiform cells. Download Figure 6-1, TIF file.

**Figure 7.**
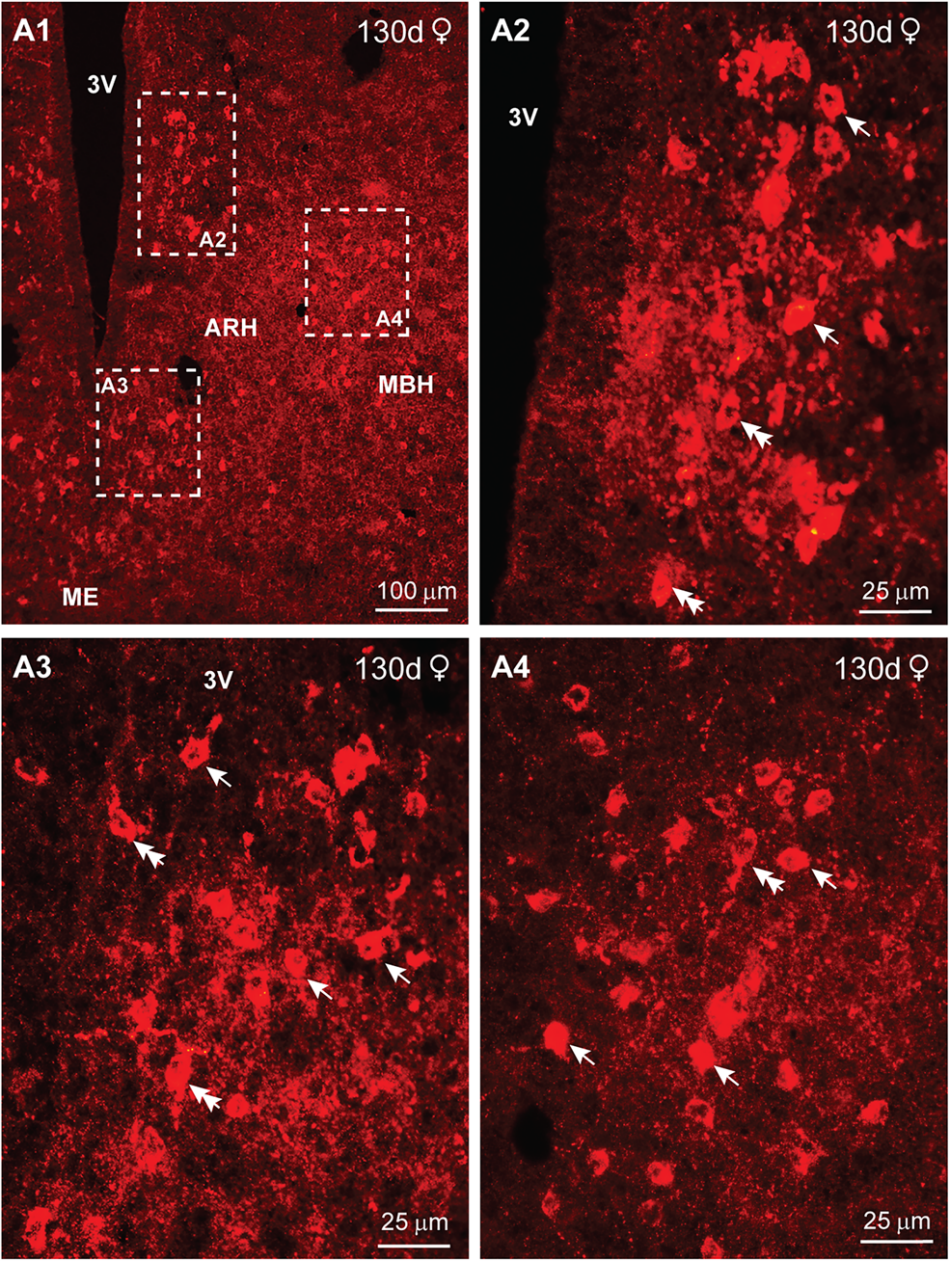
IR-βEnd expression in the fetal hypothalamus at day 130 of gestation*.* Fluorescent images of a coronal section through the medial basal hypothalamus (MBH) and arcuate nucleus (ARH) of a day 130 female fetus, illustrating the distribution of IR-βEnd in the ARH/MBH region (***A1***). High-magnification images of IR-βEnd cells along the 3V (***A2***), ventral to the 3V (***A3***), and more lateral in the MBH (***A4***) are illustrated. Scale bars show the degree of magnification. Arrows point to round cells and double arrows to more fusiform cells.

By day 130 of fetal life as illustrated in a female, the hypothalamic β-End neurons were more clearly localized in three different hypothalamic areas: one group along the ventral 3V, another group ventral to the 3V in the ARH/ME area, and the third group more lateral in the MBH part of the hypothalamus (Fig. 7*A1*). High-power images illustrated larger round cells and fusiform cells that were not as densely packed together compared with day 70 animals (Fig. 7*A2*–*A4*). A similar distribution and size of β-End neurons was also present in males at this stage of gestation.

### Immunoreactive β-End and αMSH fiber stain in the POA

Β-End fibers and αMSH fibers were first detected in the ventral POA and periventricular POA (pvPOA) in day 70 male and female fetuses. At this time only a slight fiber stain could be found. Similarly in day 130 animals, fibers but no cells were detected in the ventral POA and pvPOA. However, the density of β-End and αMSH fibers was increased at 130 d compared with 70 d fetuses.

### Development of neuropeptide Y (NPY)

**Figure 8.**
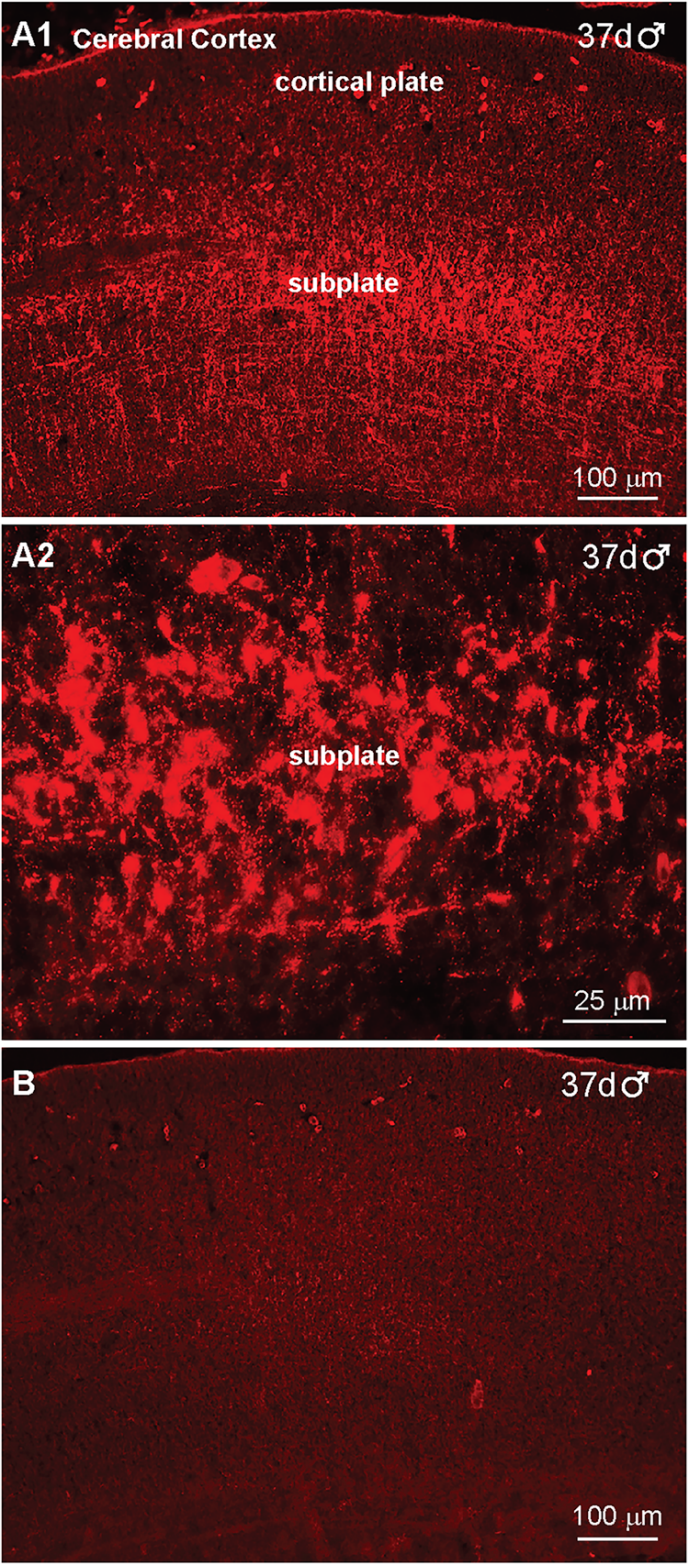
Immunoreactive Neuropeptide Y expression in the developing cortex of a day 37 male fetus. Fluorescent images of coronal sections through the subplate region of the developing cerebral cortex illustrating IR-NPY expression at day 37 of gestation (***A1***). A high-power image of cortical NPY neurons is illustrated in ***A2***. The lack of staining in an adjacent section reacted with the peptide-preabsorbed NPY antiserum (***B***). The lack of hypothalamic staining of NPY is illustrated in Extended Data Figure 8-1. Scale bars for the different images are illustrated.

Immunoreactive NPY (IR-NPY) was detected in what appears to be the outer subplate region of the developing cortex by day 37 of gestation in a fetal male (Fig. 8*A1*). Interestingly, IR-NPY cells and fibers were not detected in the BH area at day 37 of gestation or earlier (Extended Data Fig. 8-1). The high-power image, illustrated in Figure 8*A2*, clearly shows IR-NPY cells and fibers in the subplate region of the cerebral cortex at day 37 of gestation in a male fetus. Importantly, immunoreactive NPY in the cortex was eliminated when reacted with antiserum preabsorbed with the corresponding NPY peptide (see Materials and Methods), illustrating the specificity of the IR-NPY staining (Fig. 8*B*). Interestingly, it has been shown that NPY neurons are expressed in the outer cortical subplate of the developing human fetus starting at 14 weeks, and the subplate zone is an area in which IR-NPY neurons are particularly abundant (Delalle et al., 1997; Bayatti et al., 2008).

10.1523/ENEURO.0087-25.2025.f8-1 Figure 8-1 *IR-NPY was not detected in the hypothalamus in day 37 or younger fetuses*. Fluorescent image of coronal section through the diencephalon of a day 37 male fetus, that was reacted with the NPY antiserum. 3V, third ventricle; ARH, arcuate nucleus of the hypothalamus; ME, median eminence. Scale bar  =  100 µm Download Figure 8-1, TIF file.

**Figure 9.**
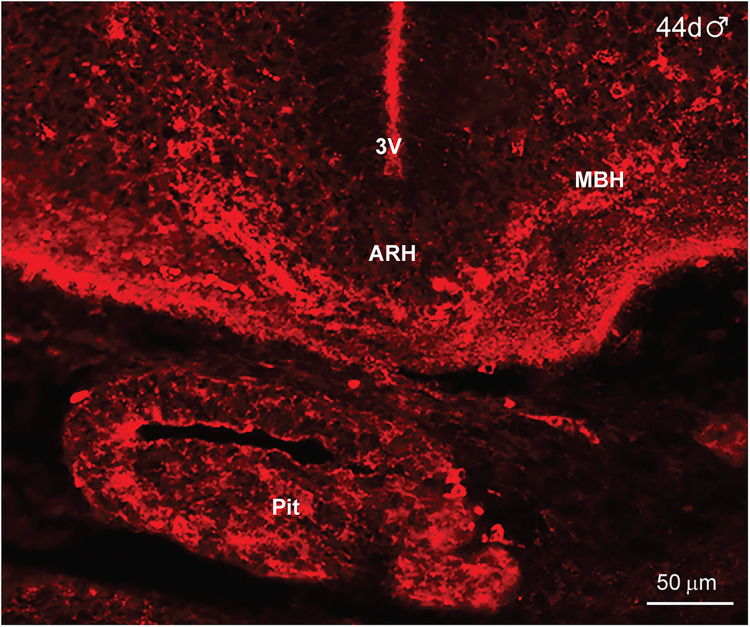
Immunoreactive Neuropeptide Y expression in the fetal hypothalamus at day 44 of gestation*.* Fluorescent high-power image of coronal sections through the hypothalamus and pituitary of a day 44 male fetus that was reacted with Neuropeptide Y (NPY) antiserum. Immunoreactive NPY (IR-NPY) is present in the ARH and MBH area and the pituitary (pit). The scale bar (50 μm) illustrates the degree of amplification.

**Figure 10.**
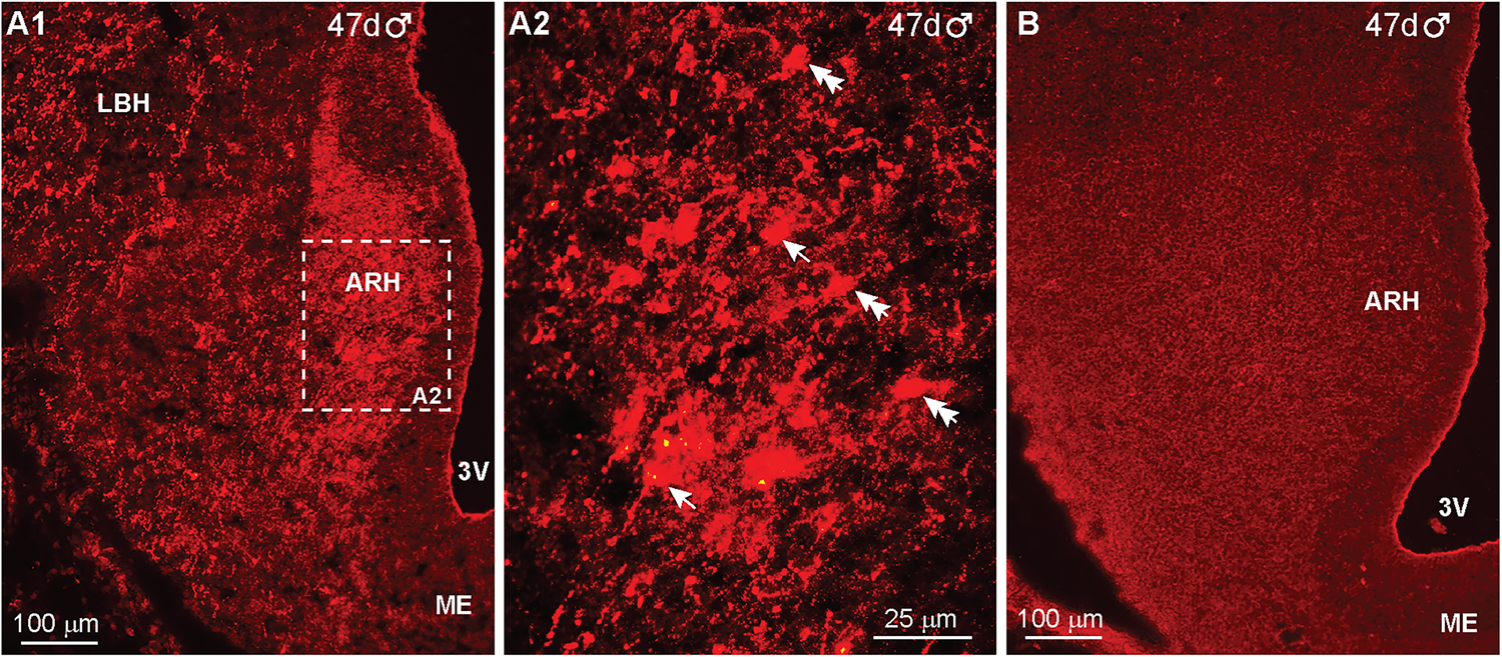
IR-NPY expression in the fetal hypothalamus at day 47 of gestation*.* Low-power (***A1***) and high-power (***A2***) images of a section through the hypothalamus of a day 47 male fetus reacted with NPY antiserum. An adjacent section reacted with NPY antiserum preabsorbed with the corresponding NPY peptide exhibited no IR-NPY (***B***). Scale bars: ***A1***, ***B***, 100 μm; ***A2***, 25 μm.

Within the hypothalamus, IR-NPY neurons were first detected at day 44 of gestation in a male fetus (Fig. 9). IR-NPY neurons were located in the ARH–MBH area, and few cells were also expressed in the pituitary (Fig. 9). The expression of cells and fibers was substantially increased (medial and lateral regions) by day 47 of gestation in another male fetus (Fig. 10*A1*). High-power images revealed round and fusiform cells (Fig. 10*A2*), and again, all hypothalamic IR-NPY was eliminated when adjacent hypothalamic sections were reacted with antiserum preabsorbed with the NPY peptide (see Materials and Methods), illustrating the specificity of the data (Fig. 10*B*).

**Figure 11.**
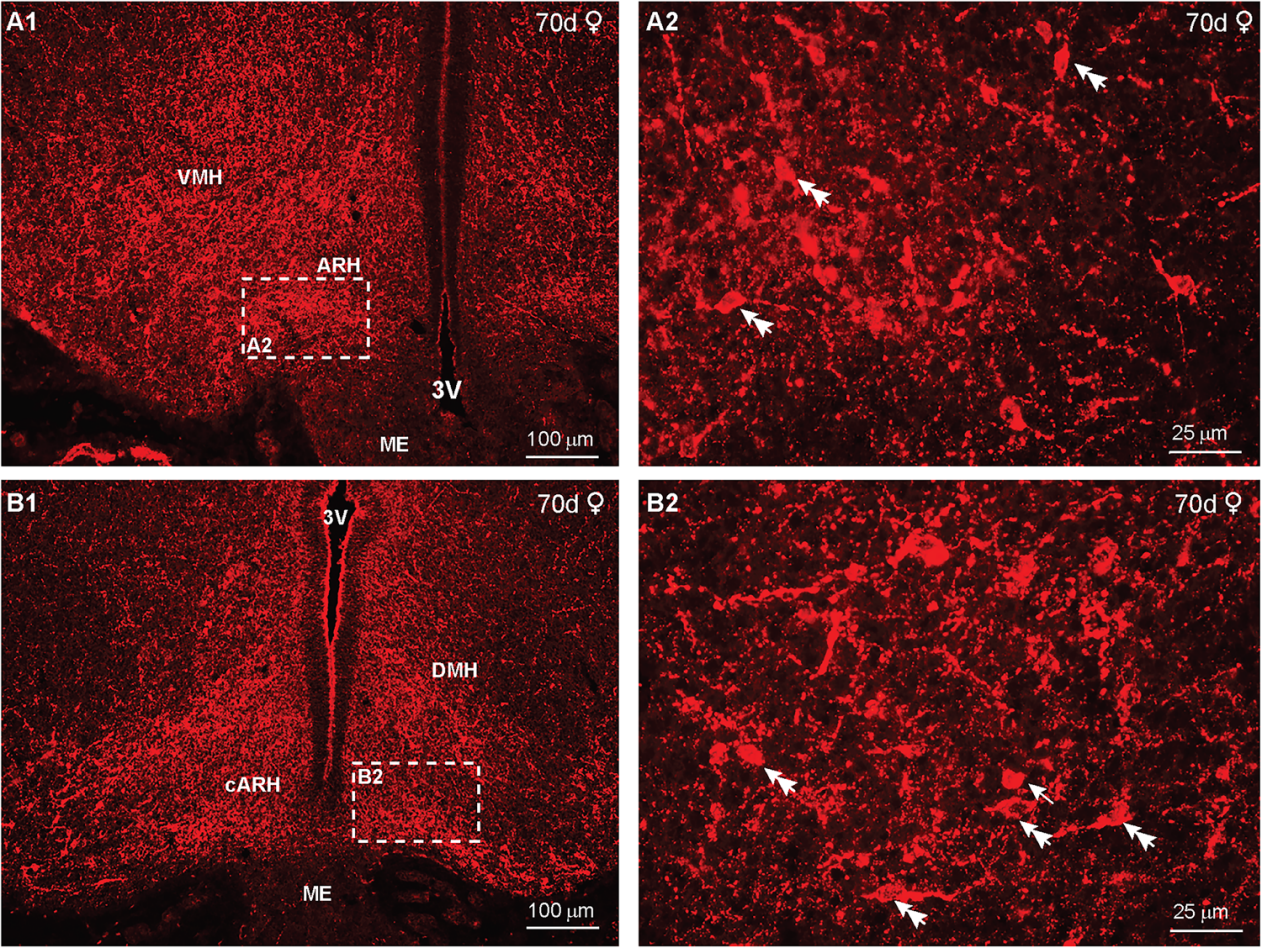
IR-NPY expression in the fetal hypothalamus at day 70 of gestation*.* Fluorescent images of coronal sections through the middle hypothalamus (***A1***) and caudal hypothalamus (***B1***) of a day 70 female fetus that were reacted with NPY antiserum. The stippled areas in ***A1*** and ***B1*** are enlarged in ***A2*** and ***B2***, respectively. The scale bars (100 and 25 μm) illustrate the degree of amplification. Arrows point to round cells and double arrows to more fusiform cells.

In day 70 fetuses, IR-NPY cells and fibers were distributed throughout most of the diencephalon, including the ARH and ventromedial hypothalamus (VMH) in a female fetus (Fig. 11*A1*). At this fetal age, NPY neurons were also present in the dorsomedial nucleus (DMH) in the caudal BH area (Fig. 11*B1*). High-power images revealed mostly round (arrow) to fusiform (double arrow) cells at this time in gestation (Fig. 11*A2*,*B2*). A similar distribution of hypothalamic NPY neurons was also found in day 70 males.

**Figure 12.**
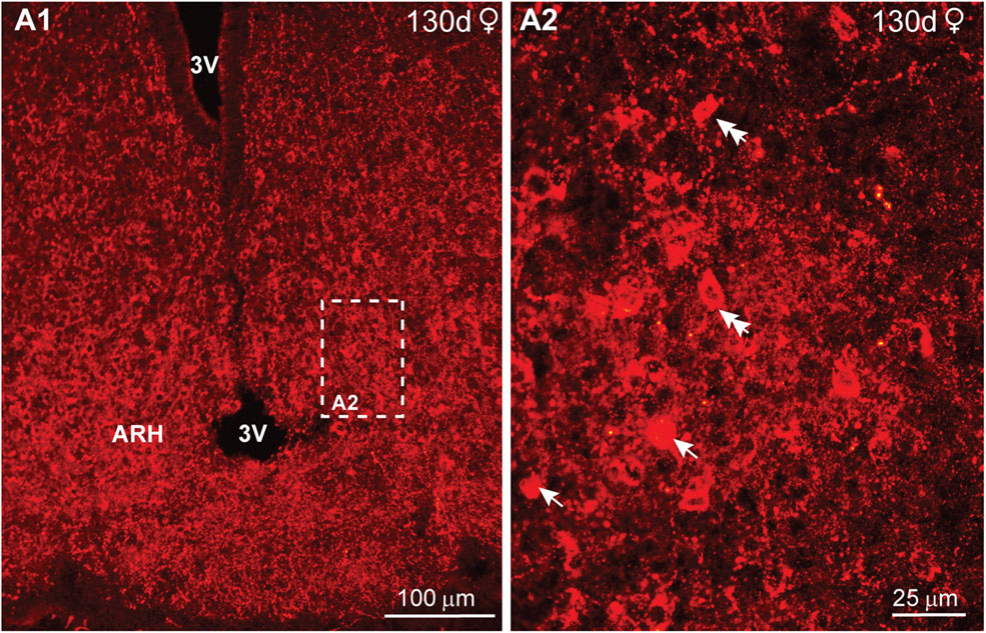
IR-NPY expression in the fetal hypothalamus at day 130 of gestation*.* Low-power (***A1***) and high-power (***A2***) fluorescent images of coronal sections through the medial basal hypothalamus (MBH) of a day 130 female fetus (***A1***, ***A2***), illustrating NPY distribution in the MBH. Scale bars: ***A1***, 100 μm; ***A2***, 25 µm.

At day 130 of gestation, the hypothalamic NPY neurons were concentrated in the arcuate nucleus, with scattered distribution in other areas (Fig. 12*A1*). High-power images revealed larger, more developed round and fusiform neurons as compared with those observed in day 70 animals (compare Fig. 11*A2*,*B2* with Fig. 12*A2*).

### Development of immunoreactive kisspeptin neurons

**Figure 13.**
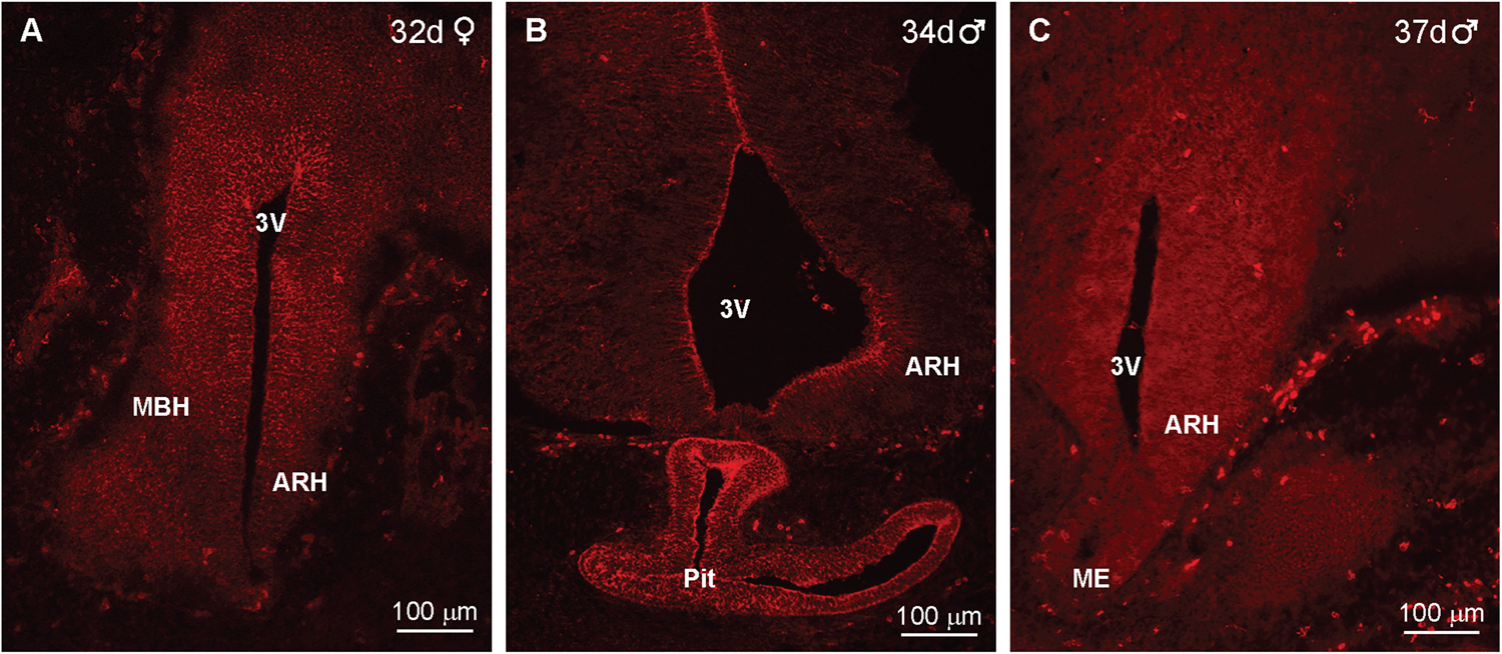
Immunoreactive kisspeptin was not expressed in the developing diencephalon or pituitary in day 37 or younger fetuses*.* Fluorescent images of coronal sections through the diencephalon of a day 32 female fetus (***A***), a day 34 male fetus (***B***), and a day 37 male fetus (***C***) that were reacted with Kiss1 antiserum. Both the brain sections (***A*–*C***) and the pituitary in ***B*** were negative to Kiss1. Scale bar: ***A***–***C***, 100 µm.

**Figure 14.**
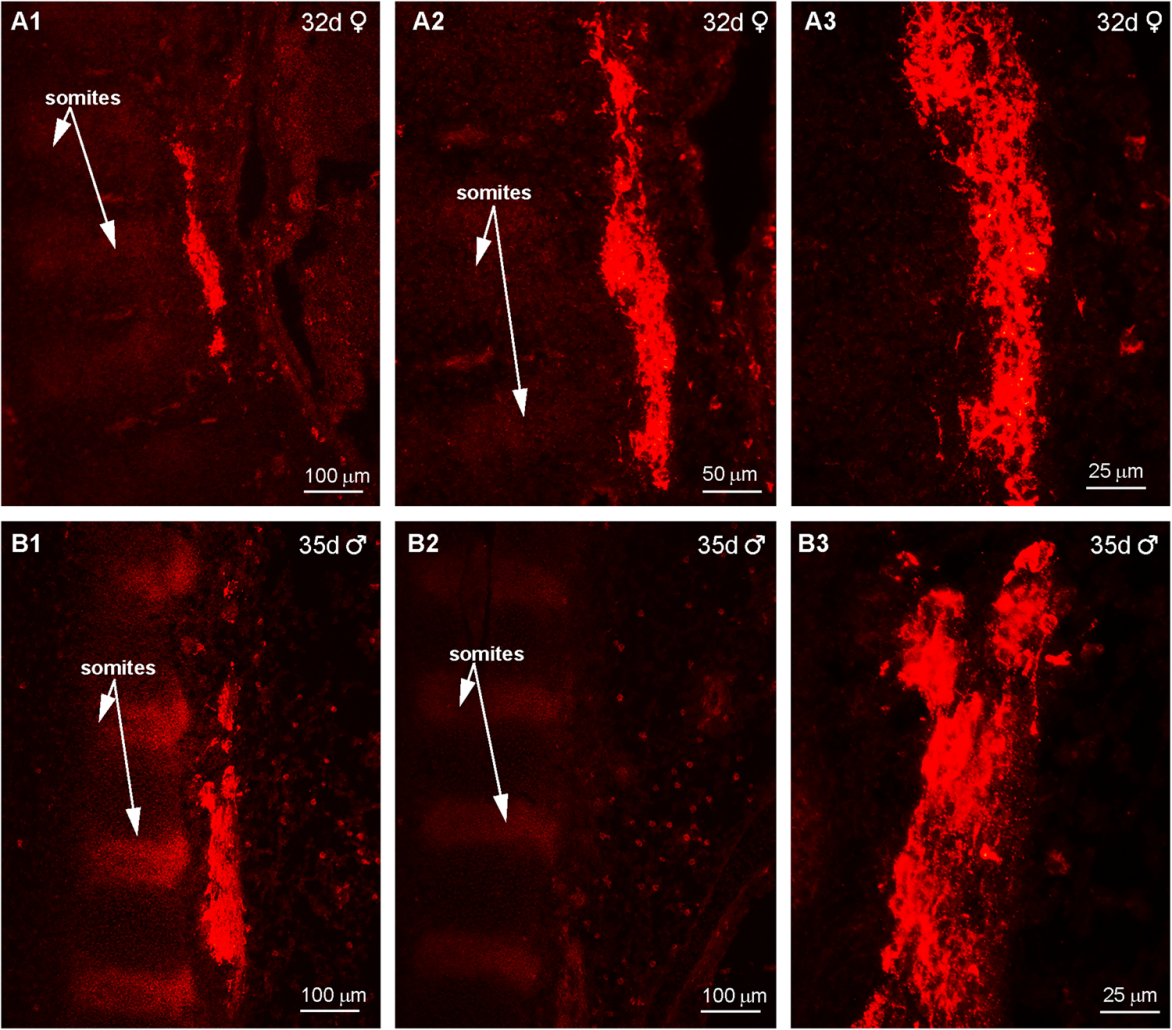
Immunoreactive Kisspeptin expression in the spinal cord of a day 32 female fetus and a day 35 male fetus*.* Fluorescent images of immunoreactive Kiss1 (IR-Kiss1) expression in the spinal cord of developing embryos beginning at day 32 (***A1***) and increased at day 35 (***B1***). Note that IR-Kiss1 is located next to the somites (vertebrates) within the spinal cord. High-power images (***A2***, ***A3***, ***B3***) illustrate that Kiss1 Cells and fibers are expressed in the spinal cord at this stage in gestation. Also, the adjacent section (***B2***) to that illustrated in (***B1***) was reacted with Kiss1 antiserum preabsorbed with kisspeptin 10, used to generate the antiserum, which eliminated the staining. Scale bars (100, 50, and 25 µm) for the different images are included to illustrate the degree of amplification.

Immunoreactive Kiss1 (IR-Kiss1) neurons within the arcuate nucleus and ventral BH developed several days later compared with POMC development. Therefore, IR-Kiss1 was not detected in the hypothalamus nor in the pituitary at days 32–37 of gestation as illustrated in a female (32 d) and two males (34 and 37 d, Fig. 13*A*–*C*). However, IR-Kiss1 was detected in the spinal cord area next to the somites (developing vertebrates) in day 32 (F) and day 35 (M) fetuses (Fig.14*A1*,*B1*, respectively). High-power images revealed IR-Kiss1 cells and fibers in the 32 d female and 35 d male fetuses (Fig.14*A2*,*A3*,*B3*). Importantly, IR-Kiss1 expression was eliminated when adjacent sections were reacted with antiserum preabsorbed with the KP peptide (listed in Materials and Methods), suggesting specificity of the Kiss1 immunoreactive staining in the spinal cord (Fig. 14*B2*). Interestingly, the presence of IR-Kiss1 in the spinal cord area appeared to be temporary, given that it was not observed in spinal cord sections from a 60 or 63 d male fetus.

**Figure 15.**
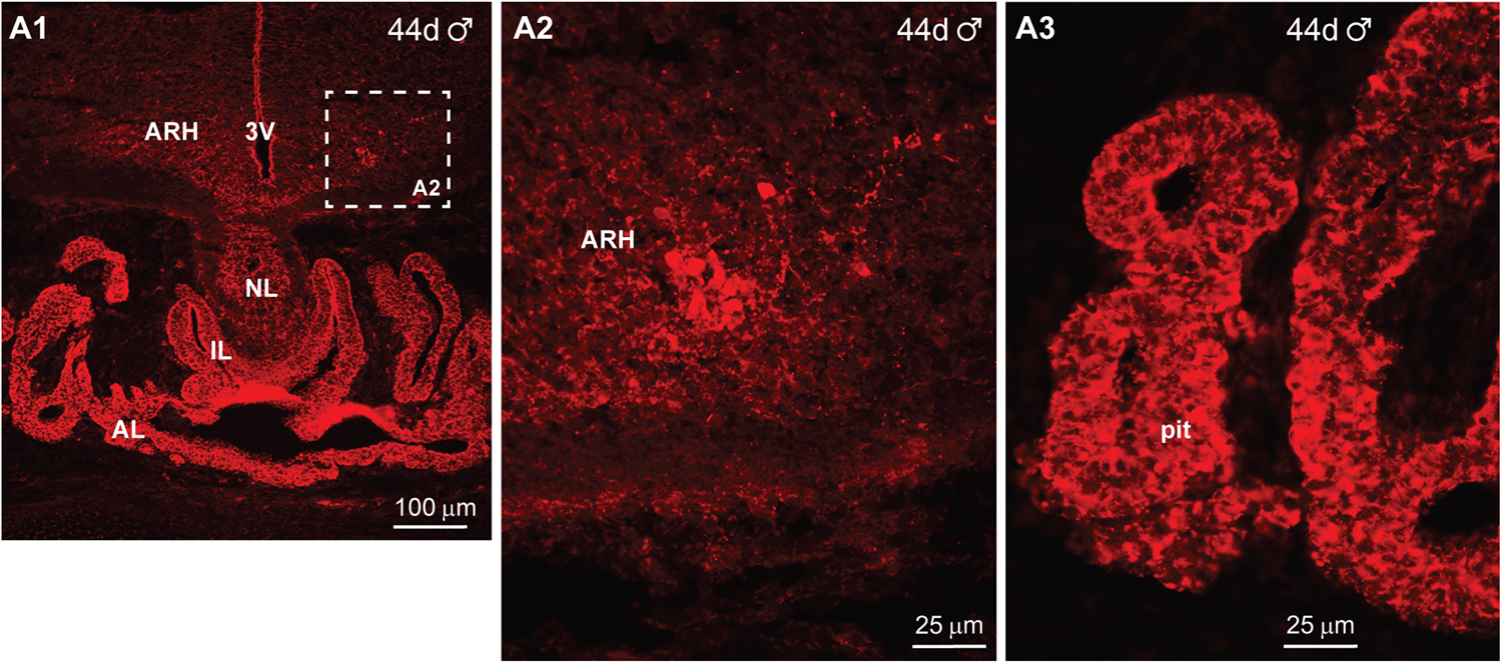
Immunoreactive kisspeptin expression in the fetal brain and pituitary at day 44 of gestation*.* Fluorescent images of coronal sections through the hypothalamus and pituitary of a male fetus illustrating IR-Kiss1 expression within the ARH at day 44 of gestation (***A***). The stippled area in ***A1*** has been enlarged in ***A2***. The high-power image (***A2***) reveals that IR-Kiss1 cells and fibers are expressed in the hypothalamus at this stage in gestation. IR-Kiss1 cells are also expressed in the anterior lobe of the pituitary (***A1***, ***A3***). Scale bars: ***A1***, 100 µm; ***A2***, ***A3***, 25 µm.

**Figure 16.**
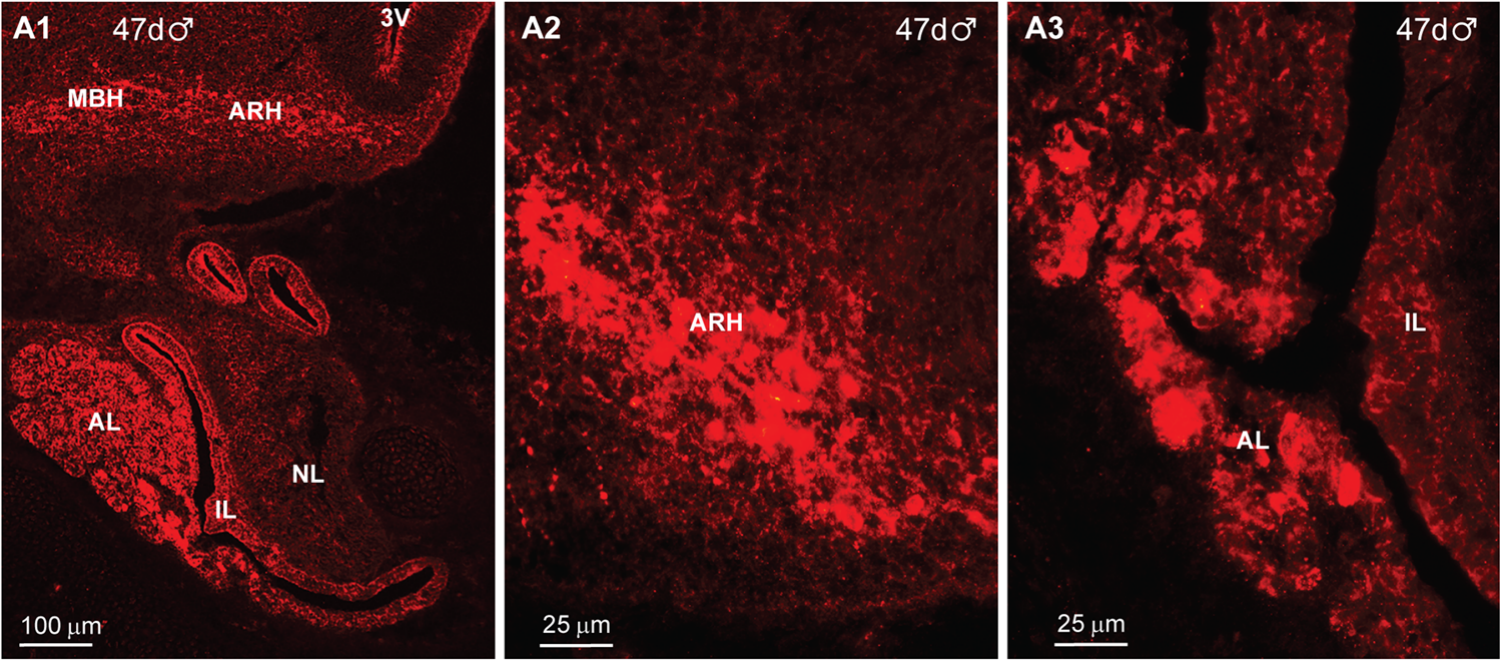
Immunoreactive kisspeptin expression in the fetal hypothalamus and pituitary at day 47 of gestation*.* Fluorescent images of coronal sections through the ARH–MBH area (***A1***, ***A2***) and pituitary (***A1***, ***A3***) of a male fetus illustrating Kiss1 expression at day 47 of gestation (***A1*–*A3***). Abbreviations: ARH, arcuate nucleus of the hypothalamus; MBH, medial basal hypothalamus. Scale bars: ***A1***, 100 µm; ***A2***, ***A3***, 25 µm.

Kisspeptin was first detected within the ARH and pituitary at day 44 of gestation in a male fetus (Fig. 15*A1*). At this time, just a few cells could be detected within the ARH in each section (Fig. 15*A1*,*A2*). Interestingly, IR-Kiss1 was highly expressed in parts of the developing pituitary at this time in gestation (Fig. 15*A1*,*A3*). The pituitary was further developed and exhibited a clear anterior lobe (AL), intermediate lobe (IL), and neural lobe (NL) by day 47 of gestation, and IR-Kiss1 was expressed in the AL and parts of the IL (Fig. 16*A1*,*A3*). In addition, the expression of IR-Kiss1 within the ventral BH and pituitary had substantially increased by day 47 of gestation and the cells were larger compared with that of the day 44 fetus (compare Fig. 15*A1*–*A3* with 16*A1*–*A3*).

**Figure 17.**
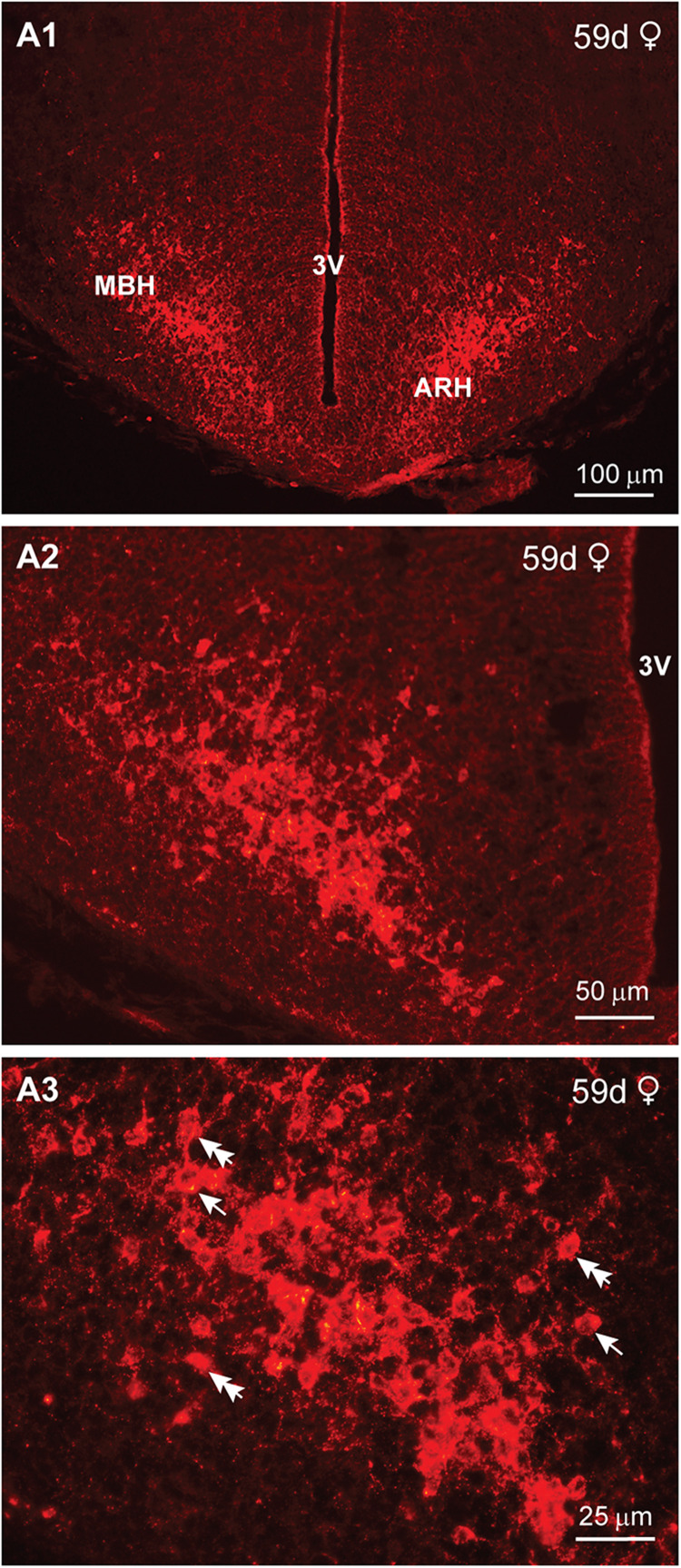
Immunoreactive kisspeptin expression in the fetal brain at day 59 of gestation*.* Fluorescent images of the coronal section through the medial basal hypothalamus (MBH) of a female fetus illustrating immunoreactive Kiss1 expression at day 59 of gestation (***A1*–*A3***). High-power images reveal round (single arrow) and fusiform (double arrow) Kiss1 cells (***A3***). Scale bars: ***A1***, 100 µm; ***A2***, 50 µm; ***A3***, 25 µm.

We next examined the expression of IR-Kiss1 at day 59 of gestation. A bilateral view of IR-Kiss1 neurons within the ARH and MBH area of a day 59 female fetus revealed an increased number of neurons at this stage of gestation (Fig. 17*A1*), and higher magnification illustrated relatively immature IR-Kiss1 neurons also at this developmental stage (Fig. 17*A2*,*A3*).

**Figure 18.**
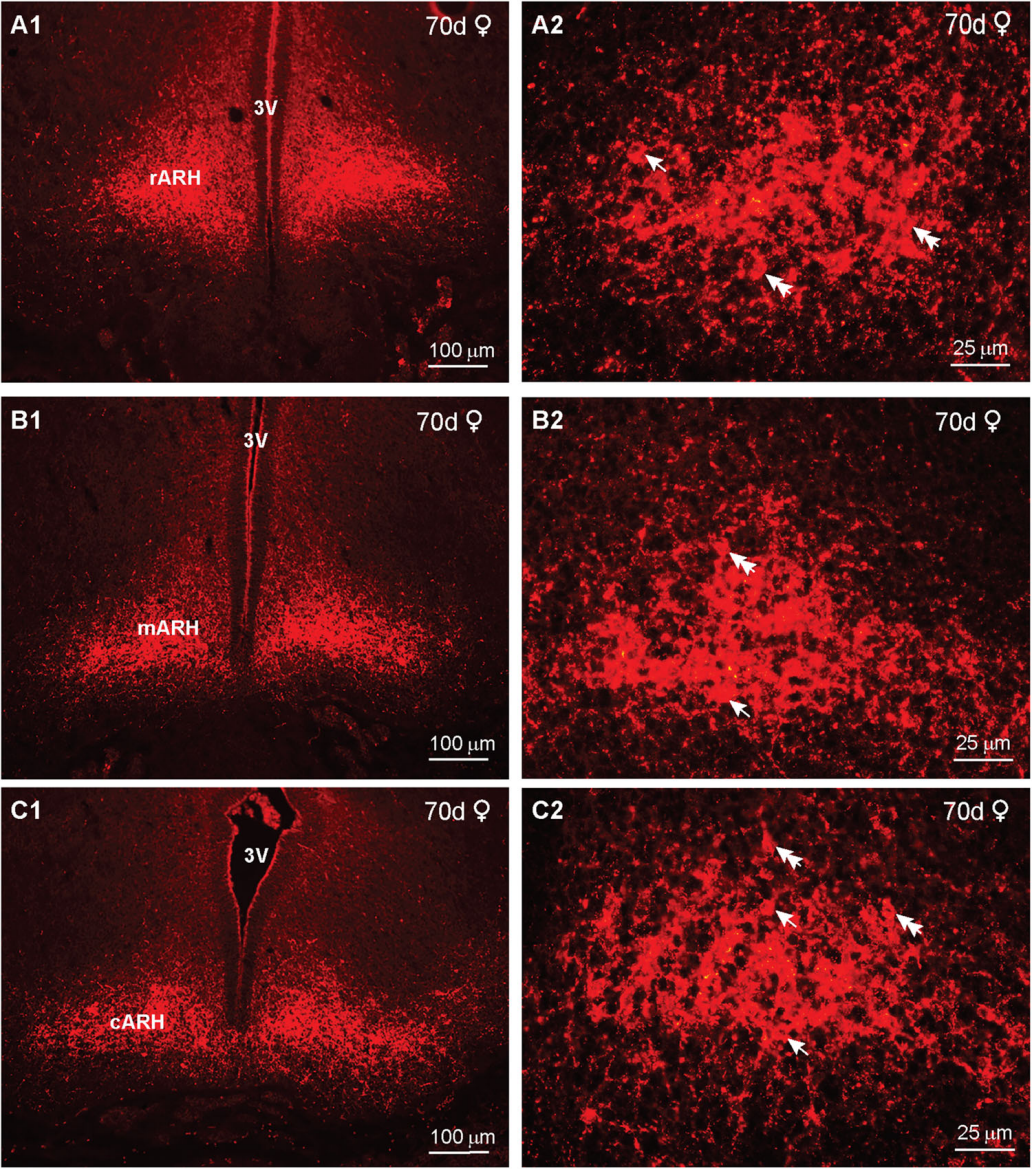
Immunoreactive kisspeptin expression in the fetal brain at day 70 of gestation in females*.* Fluorescent images of coronal sections through the rostral (***A***) to caudal (***C***) ARH of a female fetus illustrating immunoreactive Kiss1 (IR-Kiss1) expression at day 70 of gestation. The corresponding high-power images are illustrated in (***A2***, ***B2***, ***C2***). Arrows point to round Kiss1 cells, and double arrows point to fusiform Kiss1 cells. Scale bars: ***A1***, ***B1***, ***C1***, 100 µm; ***A2***, ***B2***, ***C2***, 25 µm.

In day 70 female and male fetuses, we examined the IR-Kiss1 neuronal distribution within the rostral to caudal hypothalamus. We observed a gradual increase in IR-Kiss1 from rostral to caudal in females, with the highest expression in the more caudal ARH area (Fig. 18*A1*–*C1*). The corresponding high-power images revealed a dense population of cells and fibers within each region with increasing densities toward the middle to caudal BH (Fig. 18*A2*–*C2*).

**Figure 19.**
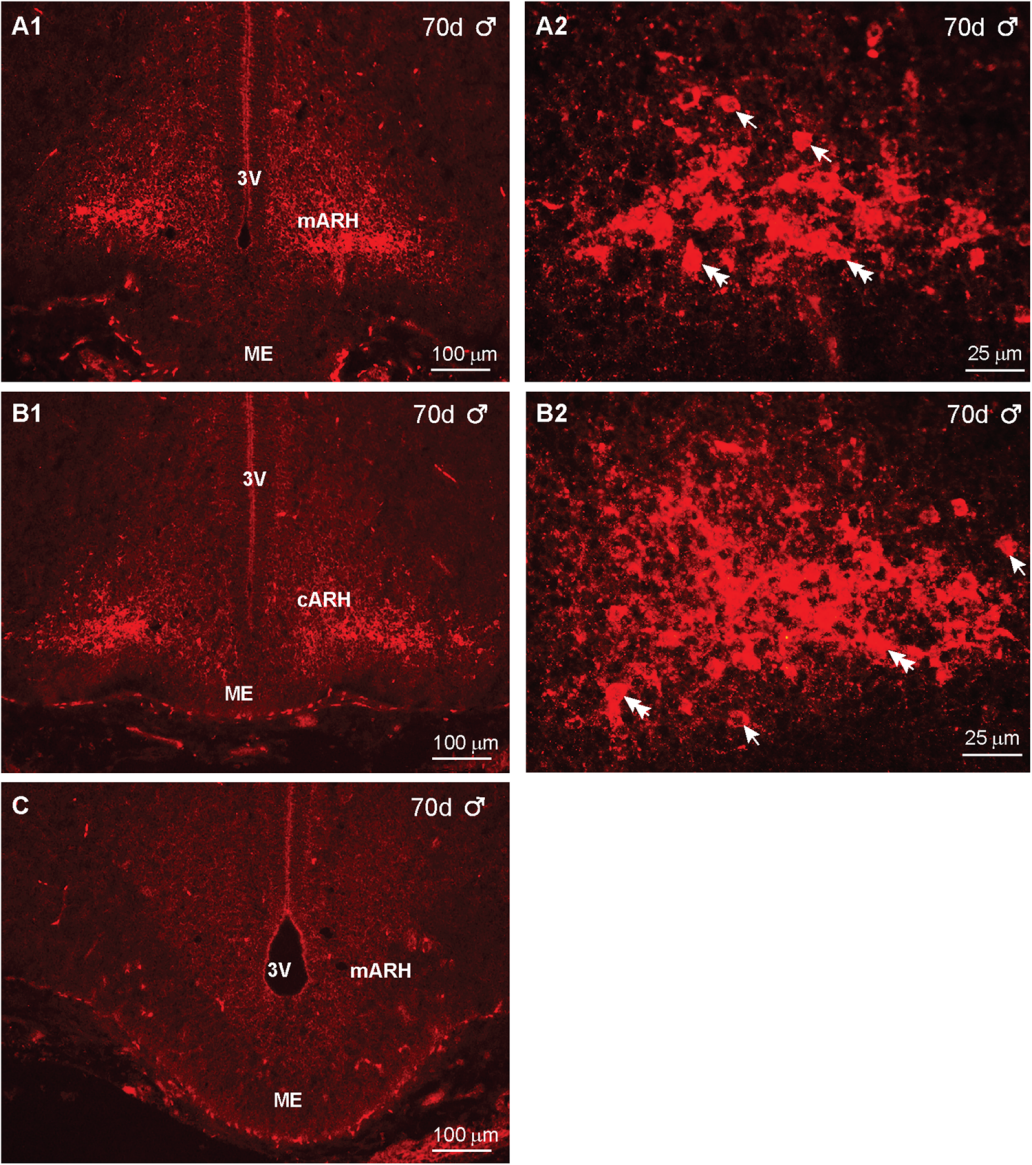
Immunoreactive-kisspeptin expression in the fetal brain at day 70 of gestation in males*.* Fluorescent images of coronal sections through the basal hypothalamus of a male fetus illustrating Kiss1 expression from middle to caudal ARH at day 70 of gestation (***A1*–*B1***). Corresponding high-power images are illustrated for each section as shown in ***A2*** and ***B2***. An adjacent section to that illustrated in (***A1***) was reacted with Kiss1 antiserum preabsorbed with kisspeptin 10, which eliminated all staining (***C***). Single arrows point to round Kiss1 cells, and double arrows point to fusiform Kiss1 cells. Scale bars: ***A1***, ***B1***, ***C***, 100 µm; ***A2***, ***B2***, 25 µm.

In comparison, we also measured the IR-Kiss1 distribution from rostral to caudal in day 70 male fetuses. Interestingly, the IR-Kiss1 distribution in the males was similar to that observed in females (Fig. 19*A1*–*B1*). However, based on high-power images, the number of cells appeared to be less, but cell size was similar: Cells identified as fusiform were 10 μm  × 8 μm in both females and males. Similarly, the round cells were 8 μm in diameter in both sexes (Fig. 19*A2*,*B2*). Importantly, a section adjacent to Figure 19*A1*, which was reacted with Kiss1 antiserum preabsorbed with the corresponding peptide (see Materials and Methods), exhibited no IR-Kiss1, confirming the specificity of the staining (Fig. 19*C*).

**Figure 20.**
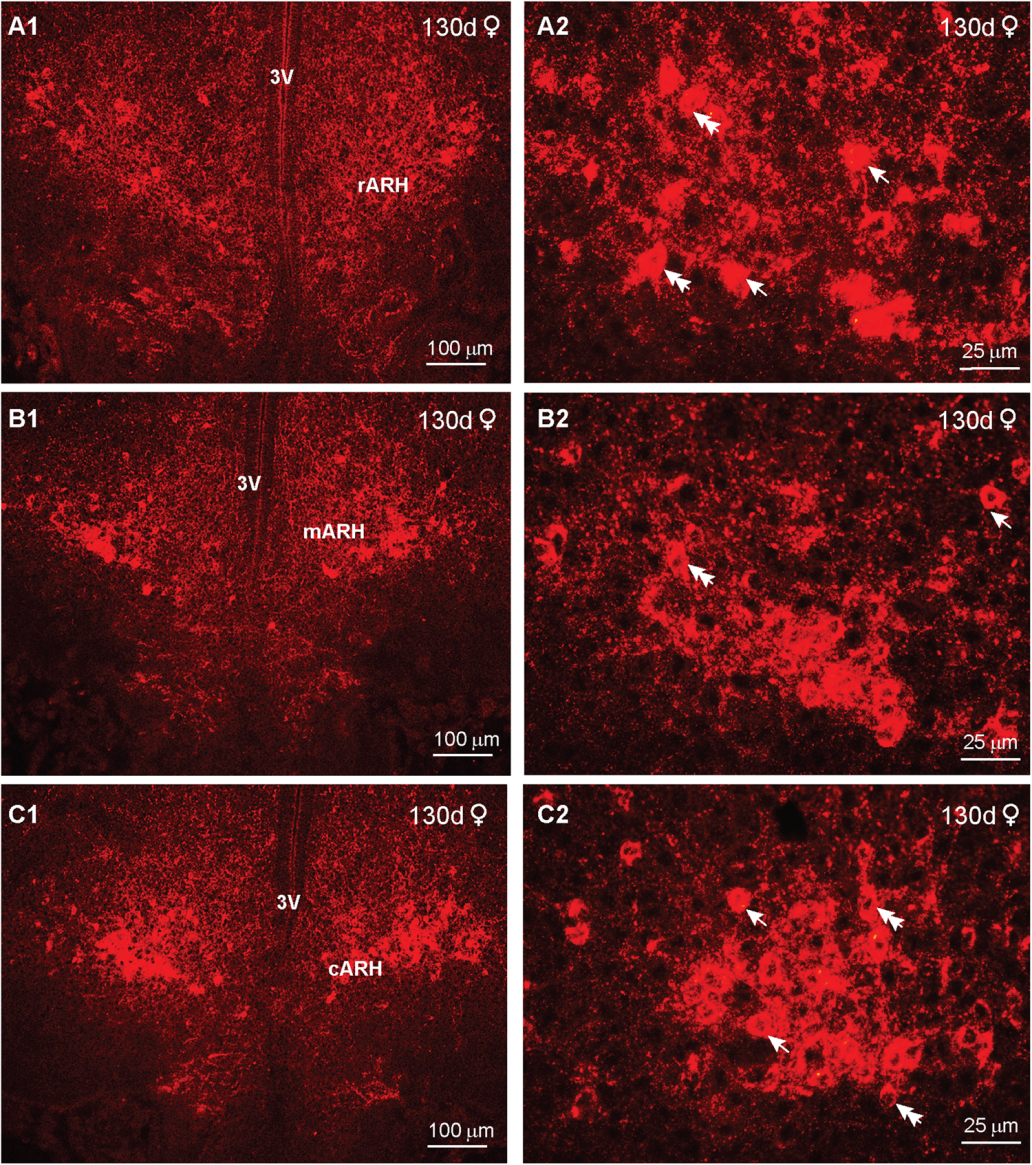
Immunoreactive kisspeptin expression in the female fetal brain at day 130 of gestation. Fluorescent images of coronal sections through the rostral (***A***) to caudal (***C***) ARH–MBH area of a female fetus illustrating immunoreactive Kiss1 (IR-Kiss1) expression at day 130 of gestation. Corresponding high-power images from the ARH are illustrated (***A2*–*C2***). Single arrows point to round Kiss1 cells, and double arrows point to fusiform Kiss1 cells. Scale bars: ***A1*–*C1***, 100 µm; ***A2*–*C2***, 25 µm.

**Figure 21.**
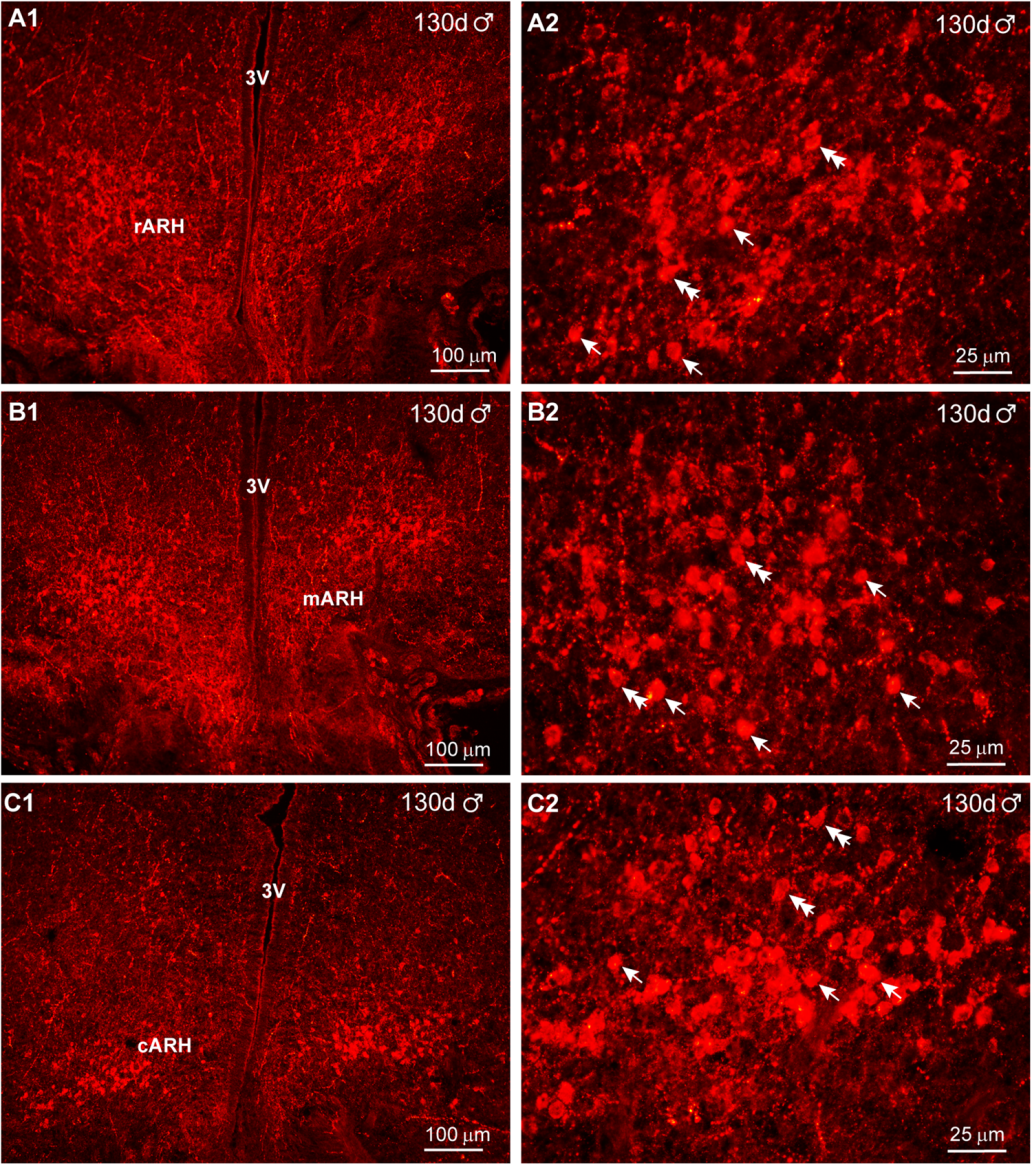
Immunoreactive kisspeptin expression in the male fetal brain at day 130 of gestation. Fluorescent images of coronal sections through the rostral (***A***) to caudal (***C***) ARH–MBH area of a male fetus illustrating immunoreactive Kiss1 (IR-Kiss1) expression at day 130 of gestation. Corresponding high-power images from the ARH are illustrated (***A2*–*C2***). Single arrows point to round Kiss1 cells, and double arrows point to fusiform Kiss1 cells. Scale bars: ***A1*–*C1***, 100 µm; ***A2*–*C2***, 25 µm.

Finally, we examined the distribution of kisspeptin in day 130 animals. At this stage in development, IR-Kiss1 neurons exhibited a wider dorsal and lateral distribution in particular within the rostral BH with more dense fiber distribution in comparison with day 70 animals (compare Fig. 18*A1*,*B1*,*C1* with Fig. 20*A1*,*B1*,*C1*). Therefore, rostrally Kiss1 cells were more spread out in both lateral and dorsal directions, whereas in the caudal BH, these neurons were more concentrated within the infundibular (arcuate) nucleus in both females and males (Figs. 20, 21). In addition, high-power images from females revealed larger fusiform (10 μm × 15 μm) and round (12 μm diameter) Kiss1^ARH^ neurons in 130 d as compared with 70 d fetuses (Fig. 20*A2*,*B2*,*C2*). Surprisingly in males, high-power images revealed slightly smaller fusiform (8 μm  × 10 μm) and round (8 μm diameter) Kiss1 neurons as compared with females at day 130 of gestation, but similar to Kiss1^ARH^ neurons in males at day 70 of gestation (Fig. 20).

**Figure 22.**
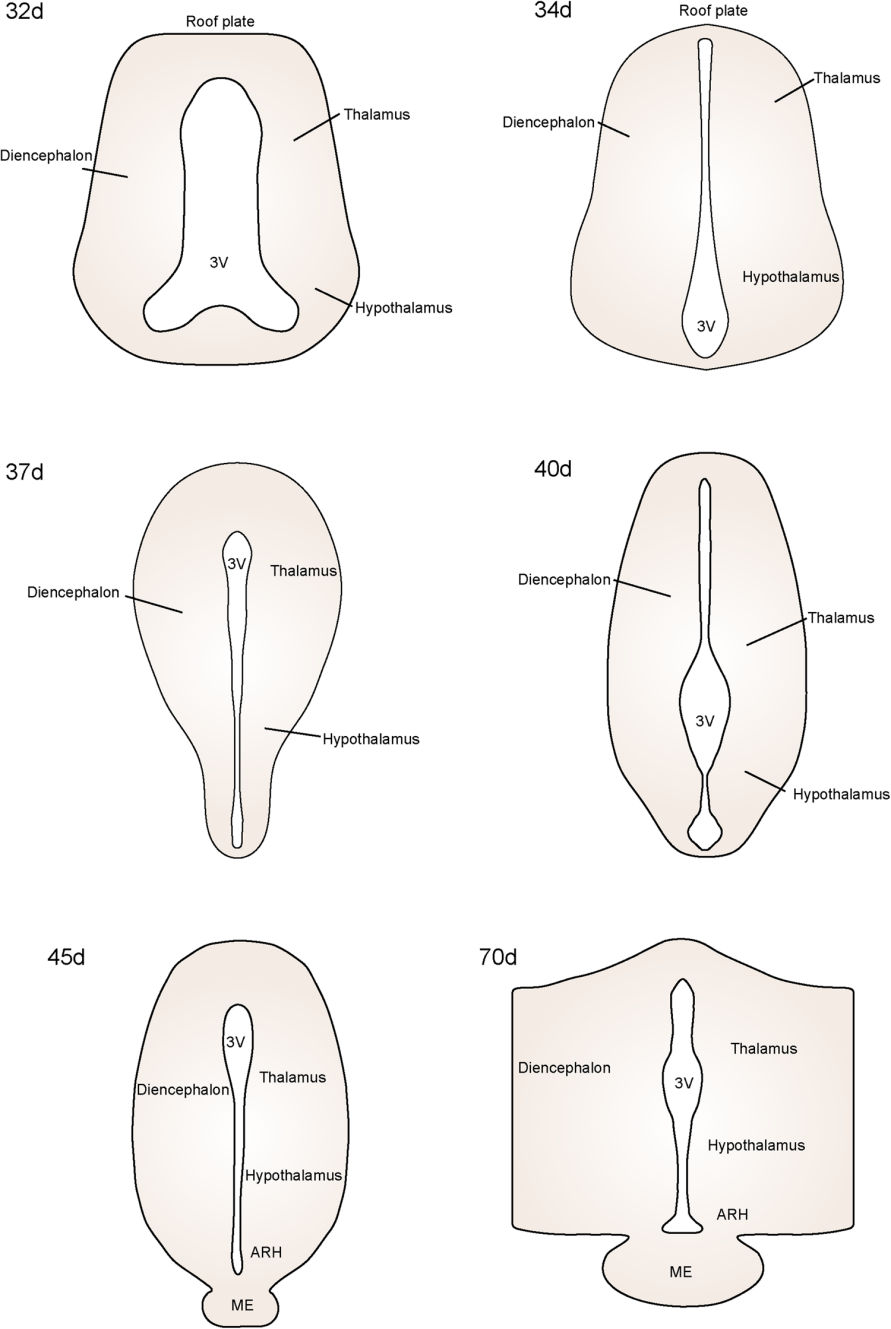
Drawings of sections through the diencephalon of days 32–70 fetal macaques. The drawings illustrate the changing shape of the diencephalon from day 32 to day 70 of gestation.

Kisspeptin was first analyzed in the fetal POA at 41 d of age and was not detected at that time. Similarly, IR-Kiss1 was also not detected in the POA of either males or females by 59–61 d of gestation**.** IR-Kiss1 fibers (but no cells) were observed in the ventral and ventrolateral POA by day 70 in females and males.

In day 130 animals, we also did not detect Kiss1 cells in the POA, with the exception of an occasional 1–2 cells in the periventricular POA area in both males and females. However, we found a more extensive Kiss1 fiber distribution in different areas in comparison with that of day 70 animals. Kiss1 fibers were present in the AH ventral to the 3V, in the periventricular area along the 3V, and extended laterally along the optic chiasm. In the dorsal POA, Kiss1 fibers extended laterally into the bed nucleus stria terminalis area.

## Discussion

We have shown the early embryonic development of hypothalamic neurons expressing the POMC peptides β-End and αMSH, as well as the neurons expressing NPY and Kiss1 in rhesus macaques, and describe the developmental progression of these neurons from day 32 to 130 of gestation. Interestingly, we found that POMC neurons are among the first to develop in the BH area, and in long-gestation primates unlike short-gestation rodents, these neurons start developing and can be identified very early in fetal life and appear to be almost fully developed by midgestation in both females and males. NPY neurons were identified in the fetal cortex at day 37 but first appeared in the ventral MBH approximately day 44–47 of gestation, which again is quite early in comparison with rodents (Padilla et al., 2010) and that described previously in primates (Grayson et al., 2006). Interestingly, prior to the expression of immunoreactive Kiss1 in the hypothalamus at days 44–47, a transient expression of Kiss1 was first detected in the developing spinal cord at day 32 and 35 of gestation, but not later in spinal cords from a day 60 fetus and a day 63 fetus. Given that these hypothalamic neurons are essential for both fertility and metabolism as described mostly in rodents, but also in primates (Hrabovszky et al., 2010; Franceschini and Desroziers, 2013; Trujillo et al., 2017; Wahab et al., 2018; Rumpler et al., 2021), it would be important to know their developmental progression and potential for functional disorders during development in rhesus macaques.

### Development of POMC neurons

Here, we show that neurons, expressing the POMC peptides β-End and αMSH, first develop in the ventrolateral diencephalon but have a connection with the dorsal diencephalon starting at day 32 of gestation. At this and later stages, cells and fibers could also be identified along the periphery of the diencephalon. Fibers along the diencephalon have been described previously in embryonic day 10.5 rodents, and these fibers were believed to be projections from hypothalamic POMC-expressing adrenocorticotropin (ACTH) neurons to other brain regions (Elkabes et al., 1989). In our study, we have documented that between day 34 and 41 of gestation in rhesus macaques, POMC neurons (β-End and αMSH cells and fibers) are located in the ventrolateral diencephalon and along the dorsal surface of the diencephalon. These neurons are first documented in the ARH/ME area by day 45 of gestation as their final destination in rhesus macaques, which is different from the findings in rodents and in Japanese macaques (Elkabes et al., 1989; Grayson et al., 2006). By day 70 of gestation, the distribution of POMC neurons within the ARH–MBH area is largely increased and similar in both males and females. Finally, by day 130 of gestation, neurons expressing β-End are located in specific regions within the ARH and MBH (Fig. 8). Interestingly, at this time, αMSH-expressing cells within the ARH are found to be similar to that of adult animals, and fibers expressing αMSH have also been documented in the PVH of the Japanese macaque (Grayson et al., 2006). Based on the early fetal development of β-End and αMSH, it is not surprising that a maternal high-fat diet can alter the developing melanocortin system and cause early-onset excess weight gain and later obesity in Japanese macaque offspring (Grayson et al., 2010; Sullivan et al., 2017).

Given that POMC peptides can inhibit feeding and metabolism via action on different receptors, it is important to know when these peptides and most likely corresponding receptors develop in fetal life (Bouret, 2022). Based on studies in mice and other rodent species, POMC neurons develop from the neural epithelium of the third ventricle approximately embryonic day 10–12 and migrate laterally (Bouret, 2022). Thus, POMC peptides first appear in basally located cells within the hypothalamus starting at day 10.5–12 of gestation and project lateral superficial fibers as well as periventricular fibers within the diencephalon (Schwartzberg and Nakane, 1982; Elkabes et al., 1989), which is different from the current findings in rhesus macaques. Specifically, the migration of neurons (cells and fibers) along the periphery of the diencephalon has not been previously described. However, the origin of these neurons in primates will need to be further explored. One similarity between primates and rodents is that POMC expression in the pituitary develops later than in the diencephalon in monkey embryos (our findings), similar to that found in mouse embryos (Elkabes et al., 1989).

### Development of NPY neurons

Currently, our studies found that NPY neurons develop later in the fetal rhesus monkey hypothalamus in comparison with POMC (β-End and αMSH)-expressing neurons. Thus, scattered NPY neurons could be detected in the ARH–MBH area first at day 44 of gestation. However, the number of NPY neurons greatly increased by day 47 and further increased in the hypothalamus by day 70 of gestation, which is much earlier than that reported previously in primates (Grayson et al., 2006). However, the staining that we observed was specific because it could be eliminated when the antibody was preabsorbed with the NPY peptide. It should be noted that also in rodents, hypothalamic NPY neurons develop later than POMC neurons. In fact, NPY neurons are derived from POMC-expressing progenitor cells in the mouse. Using molecular genetics in mice, it was discovered that POMC progenitor cells develop into NPY neurons that no longer express POMC (Padilla et al., 2010). Whether the same occurs in primates would need to be explored in future experiments. However, NPY neurons were expressed in the subplate region of the cerebral cortex already at day 37 of gestation (our study), which is an area known to express NPY later in development and postnatally in humans (Bayatti et al., 2008).

### Development of kisspeptin neurons

Similar to hypothalamic NPY neurons, hypothalamic Kiss1 neurons developed later than POMC neurons and could first be detected in the arcuate nucleus of the hypothalamus at day 44 of gestation in rhesus macaques. Interestingly, immunoreactive Kiss1 neuronal clusters appeared to be transiently expressed in the spinal cord area in the central region next to the somites already at day 32 of gestation in a female. IR-Kiss1 expression in this area was further increased in a day 35 male fetus but was not detected in the spinal cord of two males at later stages (e.g., days 60–63) in gestation. Again, the staining we observed in the spinal cord was specific because it could be eliminated when reacted with a kisspeptin antibody preabsorbed with the Kiss 10 peptide used to generate the antibody (Franceschini et al., 2006). Currently, it is unclear whether there is any connection between the transient, early expression of Kiss1 in the spinal cord and the later expression of Kiss1 in the brain. Kiss1 and its receptor (GPR54 or Kiss1R) are also expressed in peripheral reproductive tissues, such as the ovary, testis, uterus, and placenta in different species including nonhuman primates and humans, and appear to be important for follicle and oocyte maturation in females as well as spermatogenesis in males (Gaytán et al., 2009; Cao et al., 2019; Chakravarthi et al., 2021). In humans, the development of primordial follicles occurs during the third trimester of gestation, and most likely at a similar time in primates, whereas in rats this occurs postnatally (Zubeldia-Brenner et al., 2016). However, the developmental time course of Kiss1 expression in the ovaries is to our knowledge not known.

### Summary of early developmental changes of the fetal rhesus macaques

Drawings of sections through the diencephalon of days 32-70 of fetal rhesus macaques, which illustrates the changing shape of the diencephalon at this early developmental state (Fig. 22).

Hypothalamic Kiss1 neurons express both ER and androgen receptor (AR), and these neurons are important for coordinating fertility with metabolism (Franceschini et al., 2006; Kumar et al., 2015; Rønnekleiv et al., 2019; Navarro, 2020). In addition, in rodents, the POA Kiss1 neurons are essential for inducing the GnRH/LH surge, whereas it was long believed that in nonhuman primates and humans, the ARH Kiss1 neurons were responsible for pulsatile GnRH secretion and ovulation (Plant and Ramaswamy, 2009). However, recent findings suggest that E2 can upregulate Kiss1 expression in the POA in both female and male rhesus macaques as well as in humans (Trujillo et al., 2017; Rumpler et al., 2021), which is consistent with the findings that both POA and ARH kisspeptin neurons may be involved in the preovulatory GnRH/LH surges in nonhuman primates (Smith et al., 2010). Within the POA, we see only Kiss1 fibers at all stages that were evaluated up to day 130 of gestation. Therefore, the POA Kiss1 cells must appear later in gestation or postnatally in rhesus macaques.

Interestingly, the cytosolic androgen receptor is expressed quite early with the highest level at day 50 of gestation in both the male and female fetal rhesus macaque hypothalamus (Handa et al., 1988; Choate et al., 1998). Also, testosterone secreted from the fetal testes causes significantly higher levels of circulating testosterone compared with females beginning at day 60 and continuing through the rest of gestation (Resko et al., 1973; Resko et al., 1980). Importantly, testosterone secreted by the fetal testes causes sexual differentiation and male-specific behavior in′ nonhuman primates by action on steroid-binding POA neurons (Resko et al., 1980; Resko and Roselli, 1997). In addition, estradiol has been measured by radioimmunoassay (RIA) in fetal plasma of rhesus monkeys from day 59 to 163 of gestation (Resko et al., 1975). The placenta is the main source of fetal E2, and levels are low both in females and males until after 150 d of gestation (Resko et al., 1975).

At this early time, IR-Kiss1 cells are expressed in the ARH, but not in the POA of fetal rhesus monkeys (current findings). In fact, it has been shown in the embryonic male mouse that the immunoreactive androgen receptor and estrogen receptor (ERα) delineate the birthplace of kisspeptin neurons in the ARH (Kumar et al., 2015). Therefore, it is reasonable to propose that Kiss1 neurons within the ARH in both male and female rhesus macaques likely express the AR (and ER) already at days 44–50 of gestation. However, this will need to be determined in future experiments.

Currently, we found that arcuate kisspeptin neurons at days 129–130 of gestation in males were significantly smaller than kisspeptin neurons in females. Given that the testosterone from the fetal testes as described above may have stimulatory actions on POA neurons and cause sexual differentiation in nonhuman primates, testosterone could also have actions on arcuate Kiss1 neurons in males. Therefore, increasing testosterone in the circulation most likely has inhibitory actions on arcuate Kiss1^ARH^ neurons leading to the reduced size of Kiss1^ARH^ neurons later in gestation (Resko et al., 1980). Importantly, female fetuses have much lower levels of circulating testosterone together with low levels of estrogen at this developmental stage (Resko et al., 1975; Resko et al., 1980).

In summary, we have documented for the first time the early development of hypothalamic neurons expressing POMC peptides, as well as NPY and kisspeptin in rhesus macaques. Neurons expressing β-End and αMSH are the first to develop and are located in the lateral BH already at day 32 of gestation up until day 40. First at day 45 of gestation, these neurons are located in the MBH-ARH area, which is their final destination. However, these neurons change positions within the ARH/BH area as the fetus grows from day 45 to 70 and finally to day 130 of gestation. NPY neurons within the ARH develop later and can first be detected at day 44. Similarly, kisspeptin neurons develop later compared with β-End neurons and can be detected in the ARH by day 44 of gestation. Kisspeptin neurons rapidly increase in number and size from day 59 to 70 to 130 of gestation in females. In males, however, increasing levels of testosterone seem to have an inhibitory action on developing Kiss1^ARH^ neurons later in gestation resulting in the size difference of kisspeptin neurons within the ARH between males and females in rhesus macaques.

## Synthesis

Reviewing Editor: Jennifer Dulin, Texas A&M University Decisions are customarily a result of the Reviewing Editor and the peer reviewers coming together and discussing their recommendations until a consensus is reached. When revisions are invited, a fact-based synthesis statement explaining their decision and outlining what is needed to prepare a revision will be listed below. The following reviewer(s) agreed to reveal their identity: NONE.

Thank you for your patience in waiting for the review of this manuscript. The reviewer and editor agree on the novelty of this study, and only have minor comments. We strongly suggest including a visual abstract in the resubmission.

-----

Reviewer comments:

This manuscript describes embryonic development of three types of peptidergic neurons in the Rhesus monkey hypothalamus. Despite the fact that developmental changes in GnRH neurons have been reported for over 30 years, there was no report on the ontogeny of other peptidergic neurons in primates. This manuscript describes 3 types of peptidergic neurons in the hypothalamus, proopiomelanocortin peptide, neuropeptide Y, and kisspeptin, in the rhesus monkey (Macaca mulatta), a species evolutionarily close to Homo sapiens. The authors analyzed a total of 28 Macaca mulatta brains of fetal age at Day 32 to Day 130. Although the manuscript is descriptive in nature, it will greatly increase our knowledge. The following comments are minor, but incorporation will be helpful for readers.

1. The authors should consider adding schematic drawings of the brain at early fetal ages. Figures showing photomicrographs with fluorescent staining alone are often difficult to make an orientation.

2. There are many abbreviations in the text and figures. Addition of an abbreviation table including the anatomical terms, rather than abbreviations elsewhere in the text, will be helpful for the reader.

3. Table 1 needs a title. Age should be modified to fetal or embryonic age (days). What is C-R? What is head Circ? Again, the authors should not assume that all readers know those terms.

4. In the manuscript the authors repeat the gestational period of rhesus monkeys as169 days. Once the authors define the period, they do not need to repeat later again.

## Author Response

Dear Editor, Please find our responses below.

Subject: Decision on eNeuro eN-NWR-0087-25 27th Mar 2025 Dear Professor:

I am pleased to inform you that your manuscript, "Early Development of Hypothalamic Neurons Expressing Proopiomelanocortin Peptides, Neuropeptide Y and Kisspeptin in Fetal Rhesus Macaques," has been judged potentially suitable for publication in eNeuro, provided appropriate revisions are made. The decision was a result of the reviewers and me coming together and discussing our recommendations until a consensus was reached. I have put together a fact-based synthesis statement explaining the decision and appended it to this email.

Response: We are happy to hear that our manuscript entitled "Early Development of Hypothalamic Neurons Expressing Proopiomelanocortin Petides, Neuropeptide Y and Kisspeptin" has been judged potentially suitable for publication in eNeuro. I believe that we have carefully addressed all concerns by the Reviewers and Editor as outlined below.

It is important that you revise your manuscript to address the reviewers' concerns and submit a point-by-point reply to each concern. Before making a final decision, I will carefully review your revisions and responses to the reviewers.

Your revision must include the manuscript with new text indicated in a bold or highlighted font to aid the re-review of the manuscript. Separately, please provide a clean copy of the manuscript that includes the title page, which will be published if accepted. Please closely review your manuscript at this time for any final corrections in style or substance. Please consult our Revised Submission Checklist for full details on preparing your revision.

Response: New or corrected text has been highlighted.

In an effort to improve rigor, transparency, and reproducibility, eNeuro strongly encourages the use of estimation statistics. If the use of estimation statistics applies to your research, you may consider including it in your revision. More details and resources can be found in the Information for Authors.

Response: We have also included estimation statistics for increased rigor, transparency and reproducibility.

Your submission must include publication-quality figures, each in a separate EPS or TIFF (300 dpi) file. Please make sure your figures adhere to style requirements to avoid delays in manuscript processing. Detailed guidelines for figures are available in our Information for Authors.

Response: The figures have been prepared according to eNeuro recommendations.

When uploading the revised manuscript, the corresponding author will need to complete the electronic License to Publish form; if not completed during submission, a link to the form will also be available on the author's home page.

Response: The Electronic License to Publish form has been completed.

All authors are invited to submit visually appealing, high-quality images to be used to promote their article, including on the journal homepage. Please submit your images, as well as an image caption and credit information, directly to the peer review system with your revision. Additional details on sizing and image specifications can be found in the Information for Authors.

Response: This has been done.

Please return your revision within 3 months of this decision. When you are ready to submit your revision, you can log in using the link below and click on the manuscript number to create your revision. If you have any questions or concerns, or need more time to prepare your revision, please contact mailto:eneuro@sfn.org.

Yours sincerely, Jennifer Dulin Reviewing Editor eNeuro Computational Neuroscience Model Code Accessibility Comments for Author (Required):

N/A Synthesis Statement for Author (Required):

Thank you for your patience in waiting for the review of this manuscript. The reviewer and editor agree on the novelty of this study, and only have minor comments. We strongly suggest including a visual abstract in the resubmission.

Response: We are happy to hear that reviewers and editor agree on the novelty of this study and that they have only minor comments that we have addressed below.

Reviewer comments:

This manuscript describes embryonic development of three types of peptidergic neurons in the Rhesus monkey hypothalamus. Despite the fact that developmental changes in GnRH neurons have been reported for over 30 years, there was no report on the ontogeny of other peptidergic neurons in primates. This manuscript describes 3 types of peptidergic neurons in the hypothalamus, proopiomelanocortin peptide, neuropeptide Y, and kisspeptin, in the rhesus monkey (Macaca mulatta), a species evolutionarily close to Homo sapiens.

The authors analyzed a total of 28 Macaca mulatta brains of fetal age at Day 32 to Day 130. Although the manuscript is descriptive in nature, it will greatly increase our knowledge.

Response: In the revised manuscript we have included 2 additional fetal male Rhesus monkeys at days 129-130 in order to evaluate potential male female differences using statistical analysis. This was in response to the comment that the manuscript was descriptive.

The following comments are minor, but incorporation will be helpful for readers.

1. The authors should consider adding schematic drawings of the brain at early fetal ages. Figures showing photomicrographs with fluorescent staining alone are often difficult to make an orientation.

Response: We thank the reviewers for this comment, and we have added Figure 21 with schematic drawings of the fetal diencephalon from days 32 to 70 of gestation.

2. There are many abbreviations in the text and figures. Addition of an abbreviation table including the anatomical terms, rather than abbreviations elsewhere in the text, will be helpful for the reader.

Response: We thank the Reviewers for this comment, and we have added Table 1 with all abbreviations included.

3. Table 1 needs a title. Age should be modified to fetal or embryonic age (days). What is C-R? What is head Circ? Again, the authors should not assume that all readers know those terms.

Response: Again, thanks for this comment, and both the new Table 1 and Table 2 have titles. We have also defined all abbreviations used in the Tables.

4. In the manuscript the authors repeat the gestational period of rhesus monkeys as169 days. Once the authors define the period, they do not need to repeat later again.

Response: We have only repeated the gestational age of Rhesus monkeys for emphasis in the Discussion.
