# Supplementary material for: Early Development of Hypothalamic Neurons Expressing Proopiomelanocortin Peptides, Neuropeptide Y, and Kisspeptin in Fetal Rhesus Macaques
Source: eNeuro. 2025 Jun 26;12(7):ENEURO.0087-25.2025. doi: 10.1523/ENEURO.0087-25.2025 (PMC12240023; doi:10.1523/ENEURO.0087-25.2025)
Supplement: Table 2-1 — Estimation statistics comparing body weight between females and males at 130 d gestation. Download Table 2-1, DOC file. [file eneuro-12-ENEURO.0087-25.2025-s008.doc]

**Table 2-1. Estimation statistics comparing body weight between females and males at 130 d gestation.**


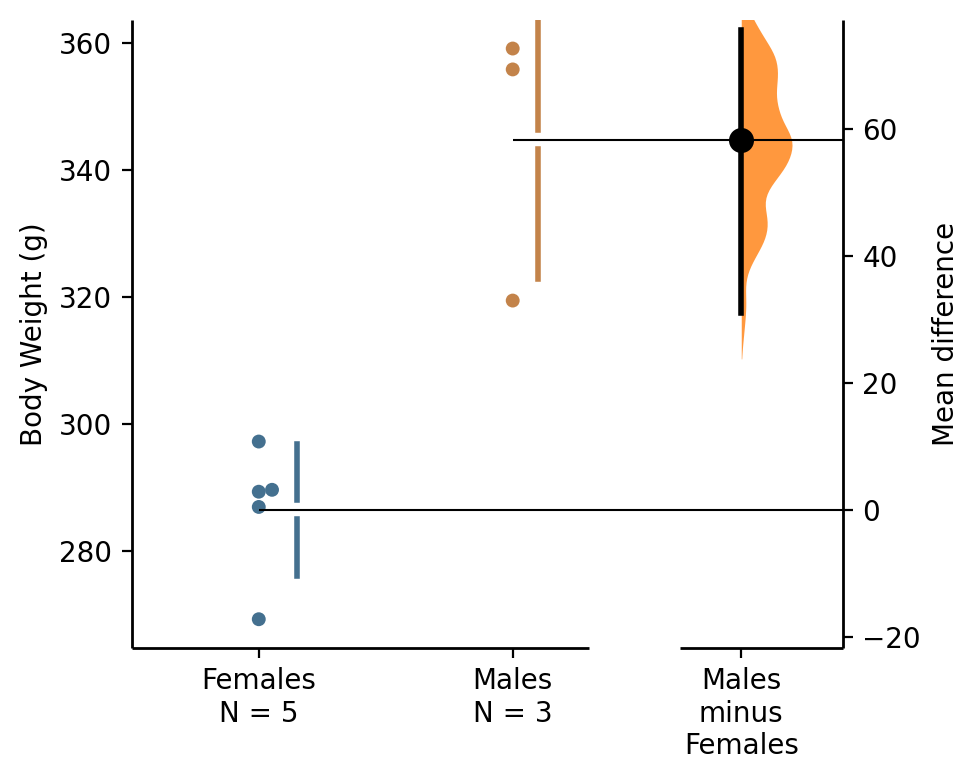


**Legend:** The mean difference between females and males is shown in the above Gardner-Altman two-groups estimation plot (Ho, et al. 2019). Both groups are plotted on the left axes; the mean difference is plotted on a floating axes on the right as a bootstrap sampling distribution. The mean difference is depicted as a dot; the 95% confidence interval (CI) is indicated by the ends of the vertical error bar.

**Results**: The unpaired mean difference between females and males is 58.3 [95.0%CI 30.9, 75.6]. The *P* value of the two-sided permutation t-test is 0.0088, calculated for legacy purposes only. The effect sizes and CIs are reported above as: *effect size* [*CI width* *lower bound*; *upper bound*]

Five thousand bootstrap samples were taken; the confidence interval is bias-corrected and accelerated. Any *P* value reported is the probability of observing the effect size (or greater), assuming the null hypothesis of zero difference is true. For each permutation *P* value, 5000 reshuffles of the control and test labels were performed.

**Reference:**
Joses Ho, Tayfun Tumkaya, Sameer Aryal, Hyungwon Choi, Adam Claridge-Chang (2019) Moving beyond P values: data analysis with estimation graphics. Nature Methods 16, 565–566.
