# Supplementary figures and images for: Early Development of Hypothalamic Neurons Expressing Proopiomelanocortin Peptides, Neuropeptide Y, and Kisspeptin in Fetal Rhesus Macaques
Source: eNeuro. 2025 Jun 26;12(7):ENEURO.0087-25.2025. doi: 10.1523/ENEURO.0087-25.2025 (PMC12240023; doi:10.1523/ENEURO.0087-25.2025)

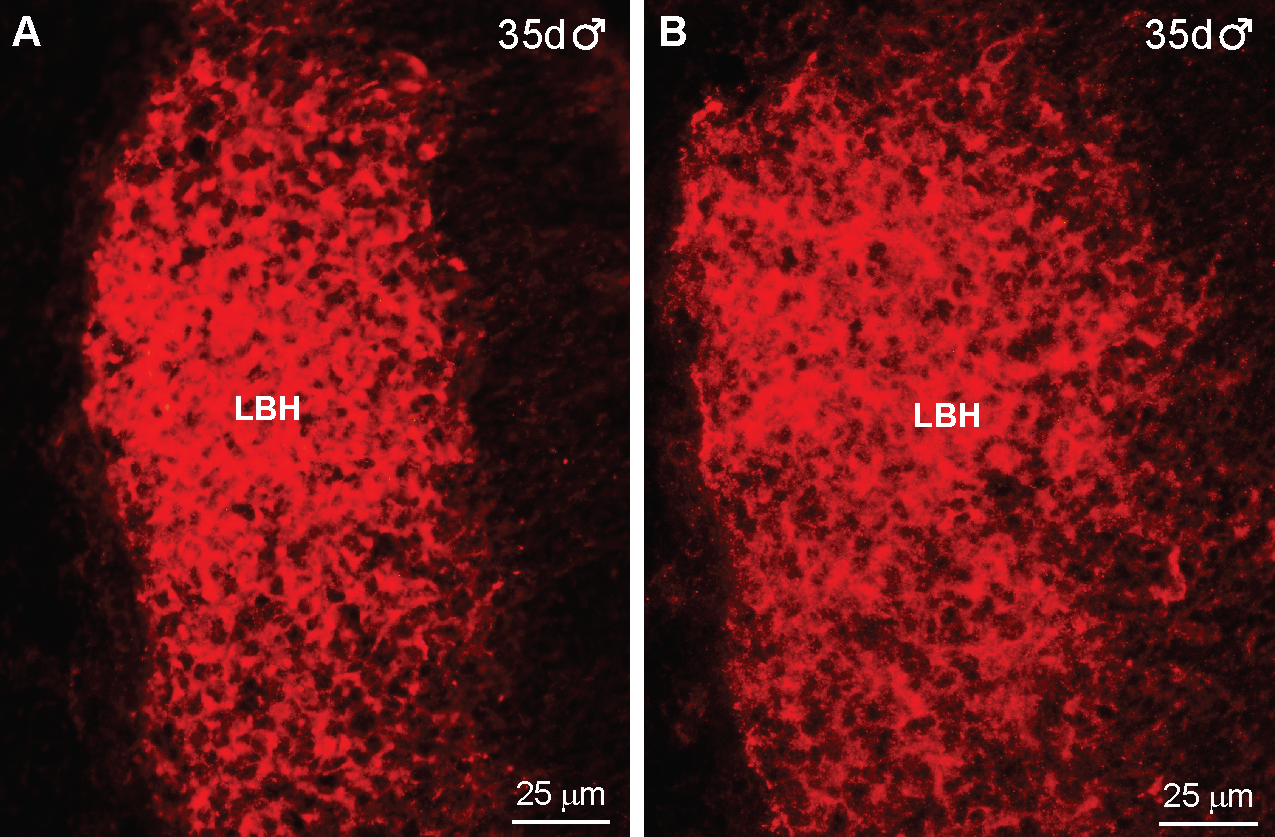

Supplement: Figure 3-1 — High power image of β-END and αMSH in the lateral BH at day 35 of gestation. Fluorescent images of coronal sections through the lateral BH (LBH) area (A, B) of a 35 day male fetus illustrating that the immunoreactive proopiomelanocortin (POMC) peptides βEnd (A) and αMSH (B) are both present at this early stage in gestation. Download Figure 3-1, TIF file. [file eneuro-12-ENEURO.0087-25.2025-s003.tif]

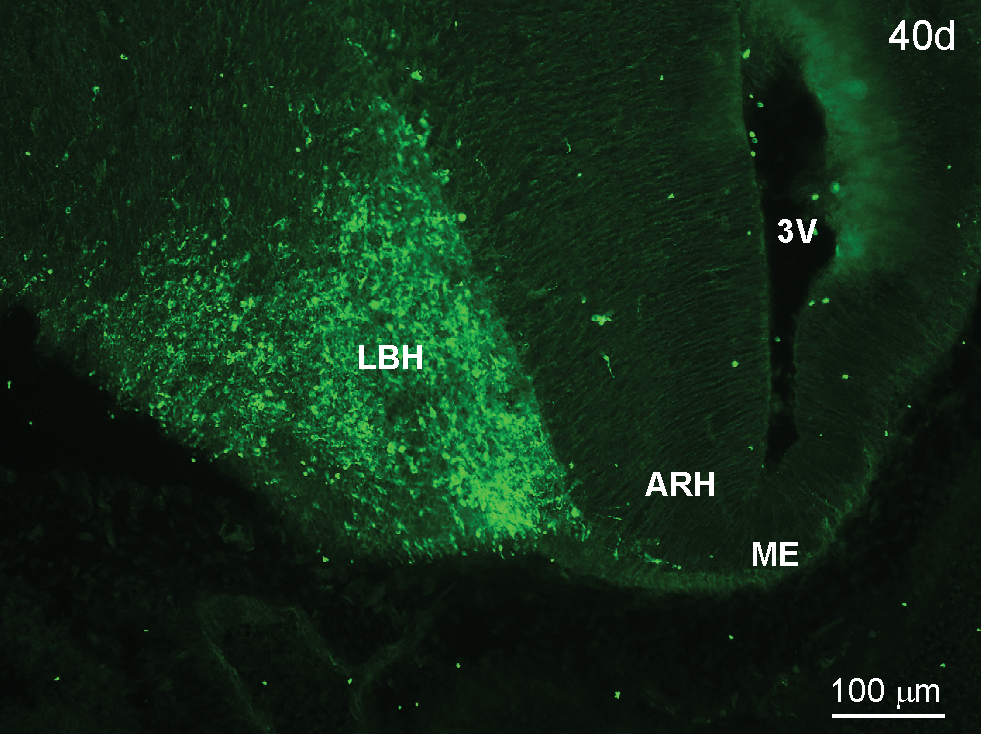

Supplement: Figure 4-1 — Immunoreactive-βEnd expression in the fetal brain at day 40 of gestation. The fluorescent image illustrates the distribution of IR-βEnd in the lateral basal hypothalamus (LBH) at day 40 of gestation. Abbreviations: ARH, arcuate nucleus of the hypothalamus; ME median eminence; 3V, third ventricle. Scale bar = 100µm. Download Figure 4-1, TIF file. [file eneuro-12-ENEURO.0087-25.2025-s004.tif]

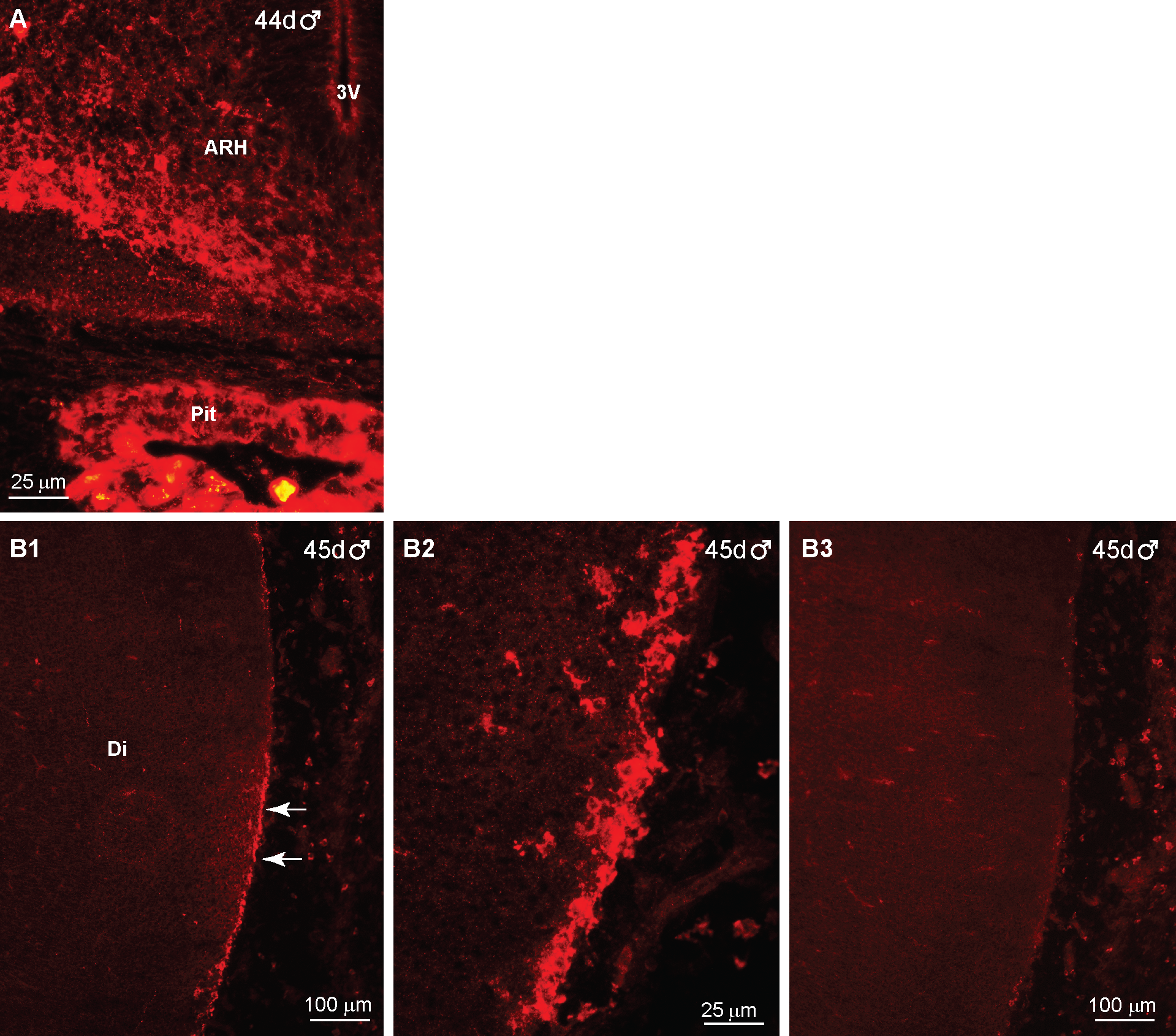

Supplement: Figure 5-1 — IR-αMSH and IR-βEnd expression in the fetal brain and pituitary at day 44-45 of gestation. Fluorescent images of coronal sections through the hypothalamus and pituitary (A), and diencephalon (Di) (B1- B3) of days 44-45 male fetuses that were reacted with αMSH (A) or βEnd antisera (B1-B2), respectively, or βEnd antiserum preabsorbed with the corresponding peptide to illustrate the specificity of the data (B3). The different scale bars are shown for all images. Download Figure 5-1, TIF file. [file eneuro-12-ENEURO.0087-25.2025-s005.tif]

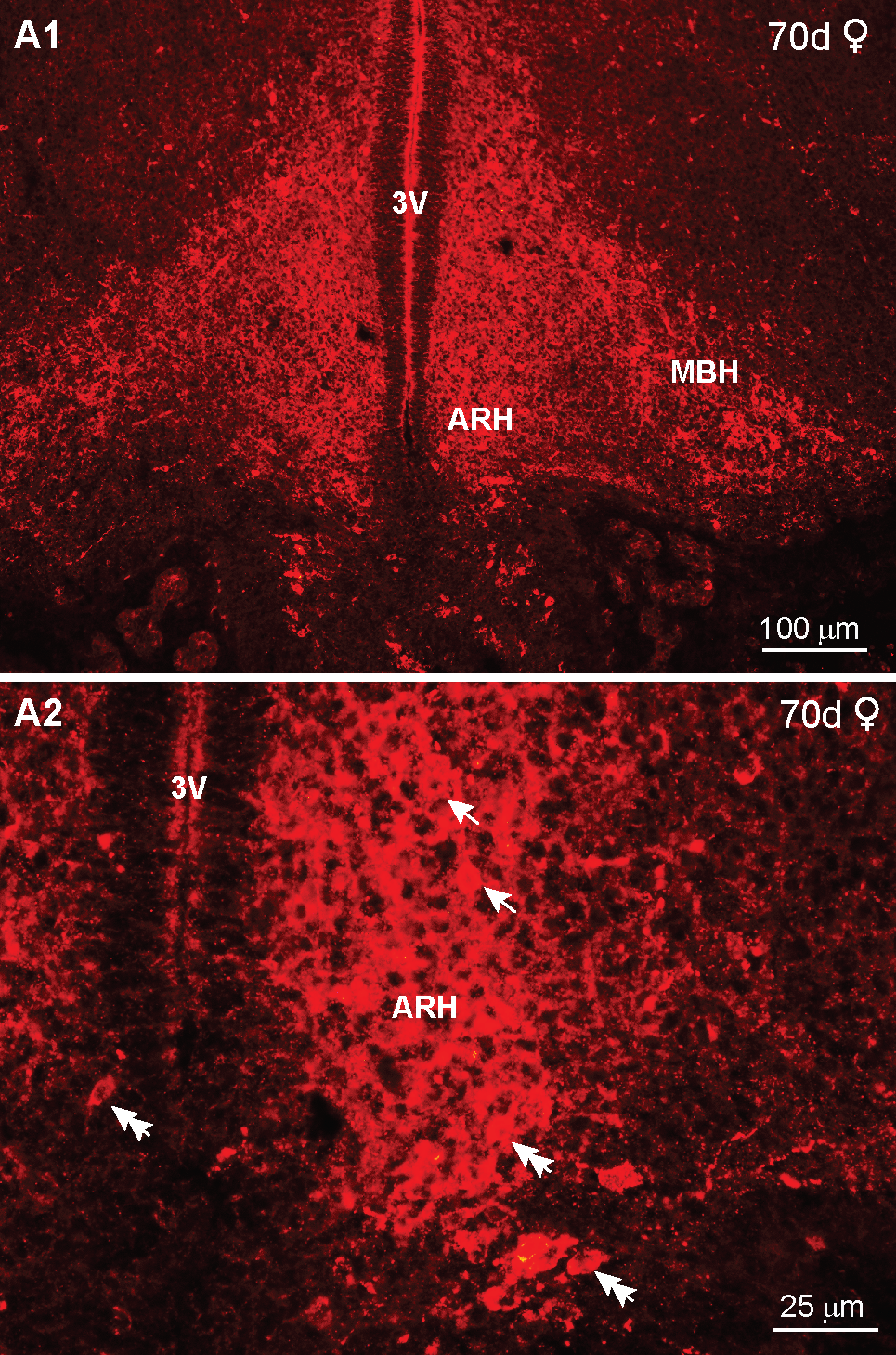

Supplement: Figure 6-1 — IR-αMSH expression in the fetal hypothalamus at day 70 of gestation. Fluorescent images of coronal sections through the BH of a day 70 female illustrating IR-αMSH within the hypothalamic area (A1). A high-power image is shown in (A2). The different scale-bars (100µm or 25µm) indicate the degree of magnification. Arrows point to round cells and double arrows to more fusiform cells. Download Figure 6-1, TIF file. [file eneuro-12-ENEURO.0087-25.2025-s006.tif]

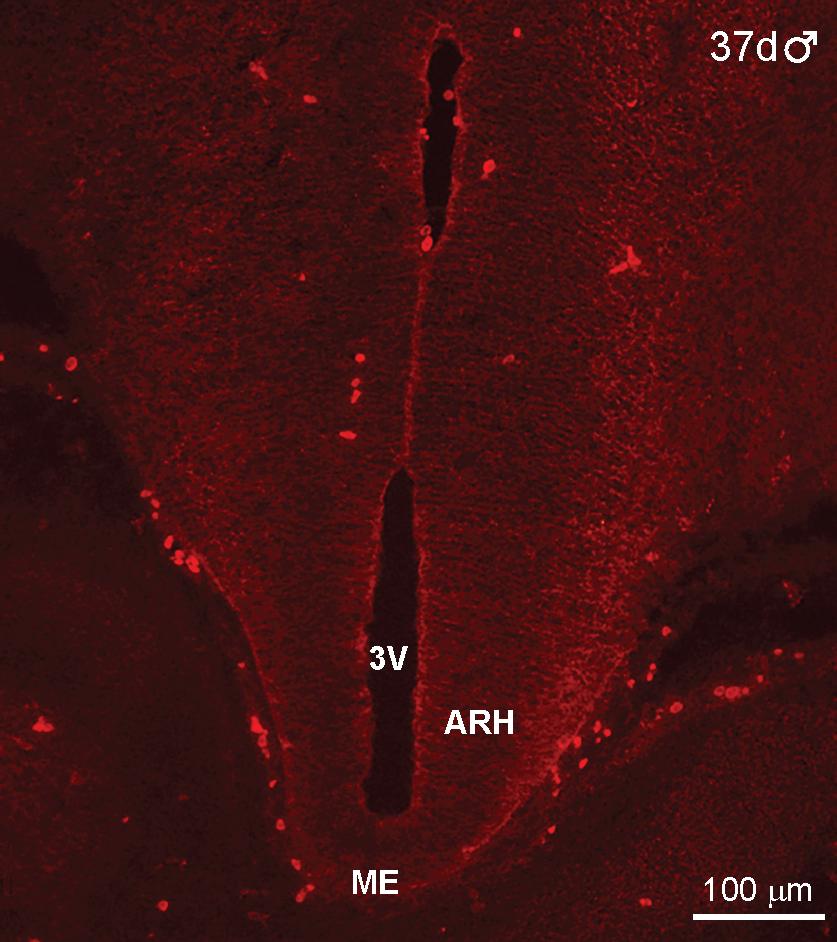

Supplement: Figure 8-1 — IR-NPY was not detected in the hypothalamus in day 37 or younger fetuses. Fluorescent image of coronal section through the diencephalon of a day 37 male fetus, that was reacted with the NPY antiserum. 3V, third ventricle; ARH, arcuate nucleus of the hypothalamus; ME, median eminence. Scale bar = 100 µm Download Figure 8-1, TIF file. [file eneuro-12-ENEURO.0087-25.2025-s007.tif]
